# Study on Contact Characteristics of Aeronautical Floating Splines Considering Maneuvering Deformation

**DOI:** 10.3390/ma19183815

**Published:** 2026-09-08

**Authors:** Yongqiang Xu, Hao Chen, Dapeng Zhang, Guangyao Hu, Hongjun Li, Kerui Xiong

**Affiliations:** 1 AECC Sichuan Gas Turbine Establishment, Chengdu 610500, China; xuyq0@outlook.com (Y.X.); chenhaodut@foxmail.com (H.C.); keruixiong@foxmail.com (K.X.); 2 China Electronics Product Reliability and Environmental Testing Institute, Guangzhou 511370, China; 3 School of Mechanics and Transportation Engineering, Northwestern Polytechnical University, Xi’an 710129, China; huguangyao@mail.nwpu.edu.cn

**Keywords:** floating involute spline, limit loads, ultimate loads, dynamic deformation, misalignment, contact stress

## Abstract

Floating involute splines are widely used in aviation power transmission systems for torque transmission. In this study, a finite element model considering the dynamic deformation of a floating involute spline shaft was established to analyze the influence of shaft deformation on the misalignment state of the spline pair under various typical dynamic overload conditions. Furthermore, a contact simulation model of the floating spline pair with an actual tooth profile was developed to investigate the effect of deformation-induced misalignment on the contact pressure distribution over the tooth surface. In addition, the contact fatigue strength of the spline pair under dynamic loading conditions, including limit loads and ultimate loads, was evaluated. The results indicate that axial overload can induce axial displacement of the mating surfaces of the floating spline, thereby reducing the effective axial contact length. Radial overload and gyroscopic moments can lead to parallel misalignment and angular misalignment of the spline, respectively. Under combined overload conditions, angular misalignment is dominant under limit loads, whereas parallel misalignment becomes more pronounced under ultimate loads. Moreover, significant stress concentration and non-uniform load distribution are observed in the contact stress field under both limit and ultimate loading conditions. A quantitative analysis method for floating spline misalignment under the superposition of multiple maneuvering loads has been established.

## 1. Introduction

In the design of aero-engine transmission systems, various types of couplings are employed for torque transmission. To improve the thrust-to-weight ratio and ensure the safety and reliability of the transmission system, couplings are required to exhibit characteristics such as lightweight construction, low cantilever moment, high-speed capability, good dynamic balance performance, acceptable centrifugal stress, and effective misalignment compensation capability [1]. Due to their compact structure, ease of installation, minimal weakening effect on shaft and hub strength, and ability to accommodate installation errors and misalignment, aerospace splines have been widely applied in aero-engine structures. For example, a single US A-4 Skyhawk attack aircraft incorporates as many as 174 splined connections [2].

However, aerospace splines operate under complex service conditions. In addition to harsh environmental influences, they are subjected to multiple mechanical loads, including centrifugal force, steady torque, periodic torque, additional cyclic torsional loads, transient peak torque, impact torque, misalignment-induced loads, and resonance [3]. These factors can lead to common failure modes such as contact wear, contact fatigue, and even structural failure during long-term operation or testing. For floating involute splines, misalignment is a key factor that causes uneven load distribution, reduced load-carrying capacity, increased contact stress, and accelerated wear and fatigue failure [2]. Statistical data from the US Navy aircraft maintenance database indicate that spline connection issues occur in approximately 40% of fixed-wing aircraft and 70% of rotary-wing aircraft [1], with misalignment-induced wear being the dominant cause. In practical operation, the coupling effects of multiple factors often result in only 25% to 50% of the spline teeth being effectively engaged. Therefore, misalignment in service splines and its influencing factors require further in-depth investigation.

Contact analysis is fundamental for evaluating the effect of misalignment in floating splines on load-carrying capacity. Therefore, numerous studies have been conducted on the contact characteristics [4,5] and load distribution [6,7] of spline joints, as well as experimental investigations on the contact pressure [8,9] and contact stiffness [10]. Regarding the contact characteristics of aerospace spline misalignment, Cura et al. [11] investigated the effect of radial misalignment angle on the load distribution between the teeth of the spline sub-tooth, and Cuffaro, Cura et al. [12] analyzed in detail the effect of the magnitude of angular misalignment and the type of lubrication of the key of the spline sub-tooth on the micromotor wear of the spline teeth of different shapes. Medina et al. [13] analyzed the contact pressure and contact stiffness of the teeth of an angular eccentric spline based on the boundary element method. Hong et al. [14] analyzed the tooth contact load distribution law of misaligned spline based on the finite element method. Zhang C. et al. [15] conducted a study on the load distribution of splines under static misalignment and dynamic misalignment conditions. Xiao L. et al. [16] studied the influence mechanism of misalignment on the wear of spline couplings. In addition, some scholars [17,18,19] have carried out theoretical modeling and research on the system dynamics of the spline coupling combined with the connected rotor. In recent years, Xu Y.Q. et al. [20] established a wear–fatigue damage model of floating splines considering the wear effect, and analyzed the distribution law of fretting fatigue cumulative damage on tooth surfaces under shaft misalignment and angular eccentricity. Zhao G. et al. [1,21,22] reviewed aviation splines and carried out design and misalignment contact characteristic studies on aviation crowned splines. The results show that under the aligned condition, the contact stress of ordinary splines is concentrated at the tooth loading end, while under the misaligned condition, the number of contacting tooth pairs of ordinary splines decreases significantly, and the contact stress increases significantly. Li J. et al. [23] established a finite element model for floating splines and analyzed the influence of different torques and different tooth side clearances on the misalignment contact characteristics of aviation splines. Iñurritegui-Marroquin et al. [24] conducted a systematic study on the working performance of spherical gear couplings (a special form of crowned spline) under high misalignment angles. Chen et al. [25] investigated the effect of crowning modification on the wear behavior of splines under different misalignment angles.

In summary, extensive research has been carried out by scholars worldwide, yielding significant progress in spline contact analysis, the effects of misalignment, and the dynamic analysis of rotating systems. However, several issues remain unresolved.

First, in (military) aero-engines, during aircraft flight, landing, catapult takeoff, and various maneuvering conditions, inertial loads can induce varying degrees of maneuver-induced deformation in floating involute spline shafts. These deformations significantly affect the misalignment state of spline pairs. At present, few studies have considered the connection state and contact fatigue strength of spline pairs under the combined influence of such complex loading conditions. Second, the use of simplified spline tooth profiles in contact analysis makes it difficult to accurately predict the distribution and magnitude of contact stress on the tooth surface.

This paper establishes a finite element model of the maneuvering deformation of a floating involute spline shaft, aiming to analyze the misalignment of floating splines under the superposition of multiple maneuvering loads. The influence of spline misalignment caused by maneuvering deformation on the distribution of tooth surface contact pressure is investigated, and the contact stress fatigue strength of the spline pair under maneuvering loads such as limit and ultimate loads is evaluated. This study provides a certain research basis for the optimal design of floating spline structures and the prediction of contact fatigue life.

## 2. Floating Spline Vice Maneuvering Load Analysis

**Figure 1:**
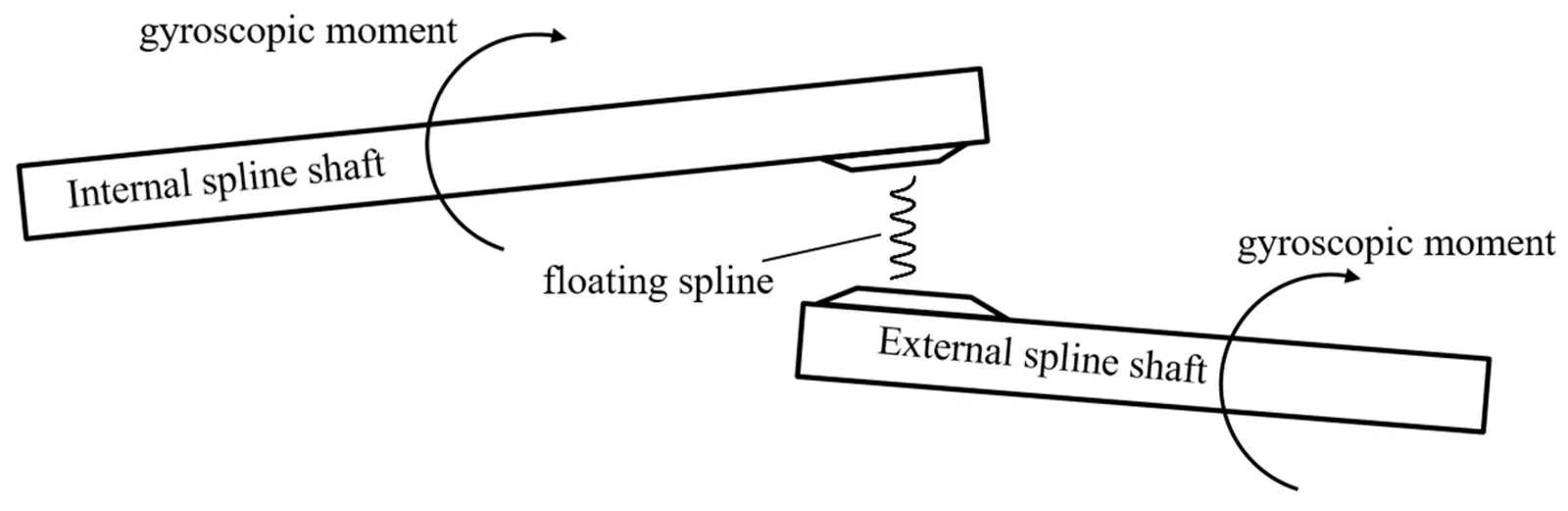
Schematic diagram of maneuvering deformation under gyroscopic moment of a typical floating spline shaft of an aero-engine.

Maneuvering loads refer to the inertial forces acting at the center of mass of engine components, the inertial moments about the engine center of mass, and the gyroscopic moments of rotating components, which arise during aircraft operations such as flight, landing, catapult takeoff, and various maneuvering conditions. Under the combined action of these loads, the floating spline shaft undergoes maneuver-induced deformation, resulting in misalignment in the mating state of the spline pair. Figure 1 illustrates the variation in the mating state of the floating spline under gyroscopic torque.

This study primarily investigates the influence of maneuvering loads, including inertial forces and gyroscopic moments, on the misalignment behavior of floating spline pairs. The maneuver-induced deformation of the floating spline shaft under such loading conditions is calculated and analyzed. Subsequently, the resulting misalignment of the spline pair under different maneuvering loads is extracted, and its effect on the contact stress distribution of the spline pair is further examined.

**Figure 2:**
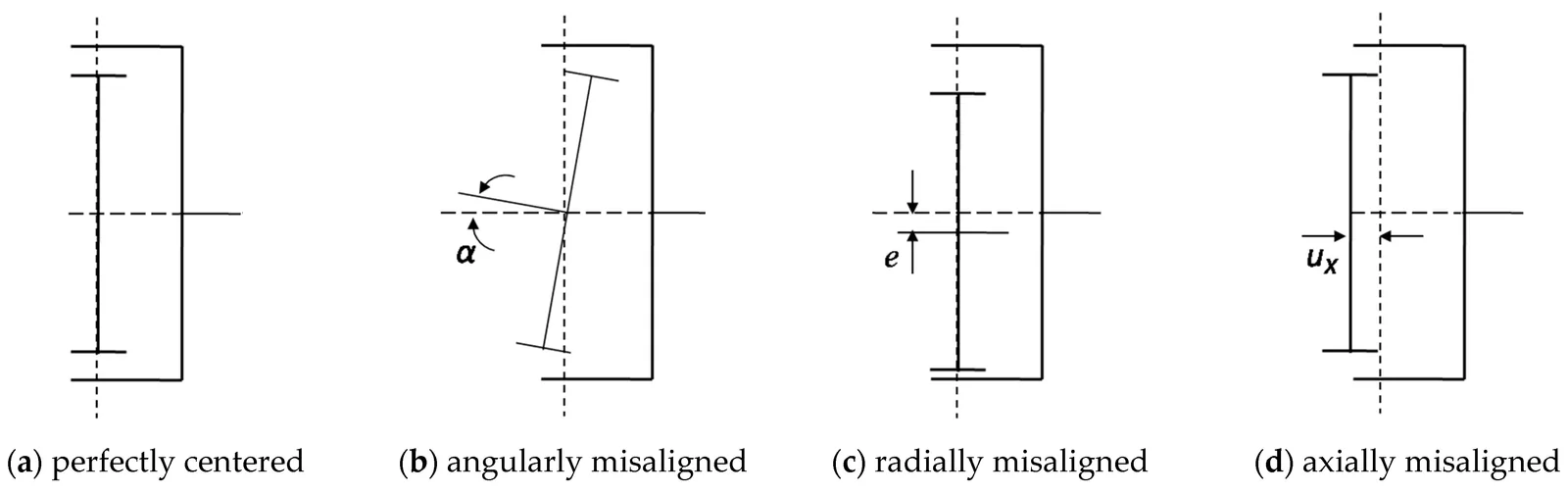
Schematic diagram of misalignment of floating spline pair.

**Figure 3:**
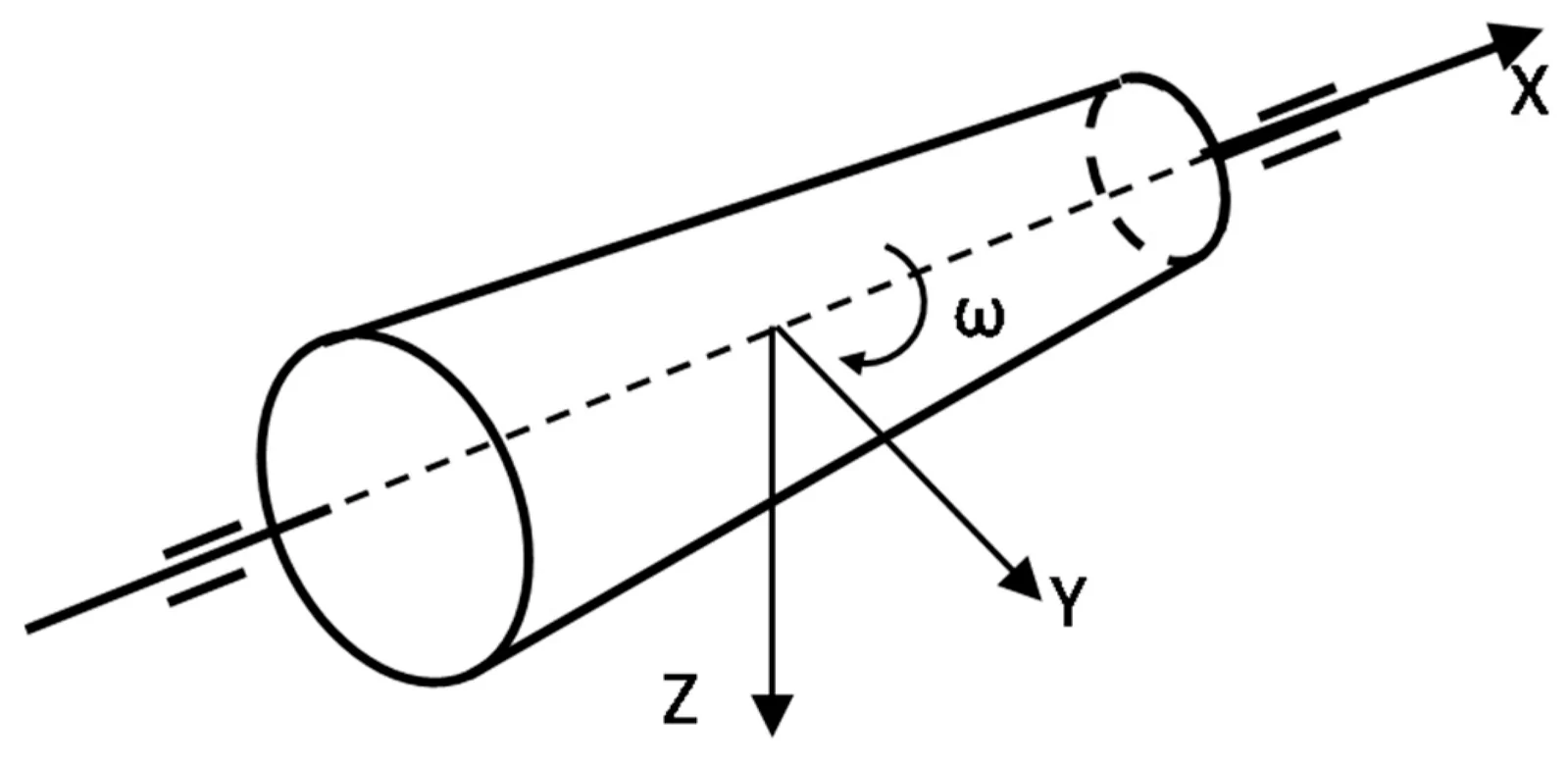
Cartesian coordinate system for motorized load analysis of floating spline shafts.

**Figure 4:**
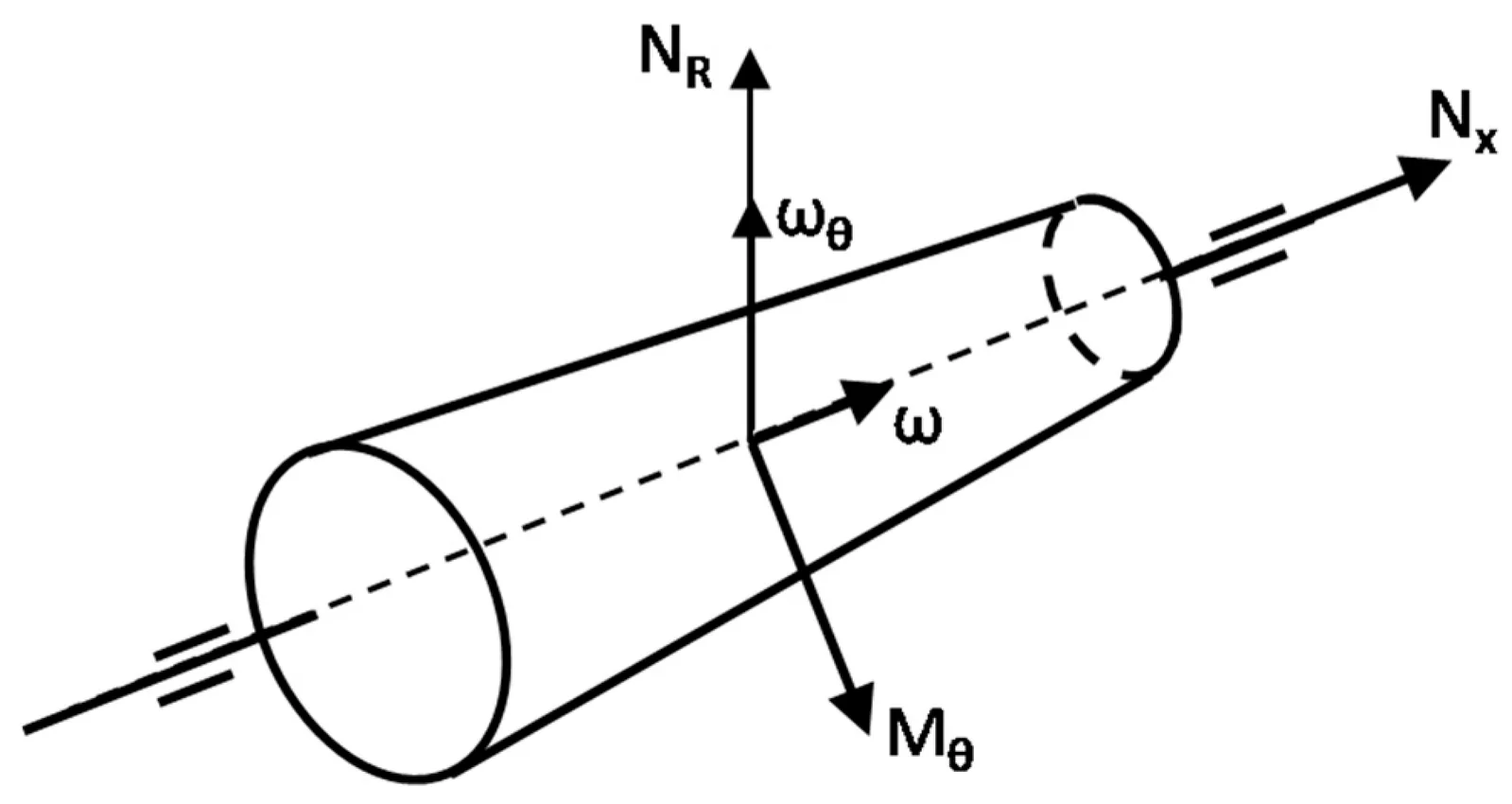
Schematic diagram of floating spline shaft motorized loads.

The misalignment types of a floating spline pair can be categorized into three cases: angular misalignment, radial misalignment, and axial misalignment, as illustrated in Figure 2. The corresponding symbols defining the misalignment magnitudes are given in Table 1. Figure 3 depicts the Cartesian coordinate system employed for analyzing the maneuver loads on the floating spline. The X-axis coincides with the rotational axis of the spline shaft, with the positive direction defined from the internal spline towards the external spline. The Z-axis is oriented vertically downward (positive downward). The Y-axis is determined by the right-hand rule, oriented positively to the right along the X-axis. ω denotes the rotational angular velocity of the spline shaft. The inertial force overloads acting on the floating spline are decomposed into axial, vertical, and horizontal directions. Since the vertical and horizontal overloads exert identical influences on floating spline misalignment, they are consolidated herein as the radial overload. Similarly, the gyroscopic moments of the spline shaft are decomposed into vertical and horizontal yaw components, which also exhibit equivalent effects on spline misalignment; consequently, they are combined into a single yaw rate. Synthesizing the above considerations, the defined overload types for the floating spline and their corresponding symbols are presented in Table 2. Figure 4 provides a schematic representation of the maneuver loads acting on the floating spline. It is crucial to note that the vector directions of the radial overload and the gyroscopic moment are aligned, resulting in the most hazardous superimposed misalignment effect under this condition. The calculation equations for the mass inertial force and gyroscopic moment in Table 2 [26] are shown in Equations (1)–(3):

(1) $$F_{X}=mgN_{X},$$

(2) $$F_{R}=mgN_{R},$$

(3) $$M_{\theta }=J\omega \;\times \;\omega_{\theta },$$

where *m* is the spline shaft mass; *g* is the gravitational acceleration; *J* is the spline shaft moment of inertia.

During flight, landing, catapult takeoff, and various maneuvering flights of an aircraft, there are situations where multiple maneuvering loads are superimposed. Table 3 presents the maneuvering overload superimposition coefficients under typical working scenarios of the floating spline, which refers to the relevant requirements for maneuvering overload in China’s relevant standards [27] and is mainly divided into two cases:

(a) The capability of the floating spline to operate under short-term conditions combining ±3.5 rad/s yaw angular velocity and 1 g radial overload is defined as the ultimate load scenario.
(b) The capability for long-term operation under conditions combining ±1.4 rad/s yaw angular velocity with either 10 g radial overload or 5.5 g axial overload is defined as the limit load scenario.

## 3. Spline Misalignment Analysis Method

### 3.1. Calculation of Spline Pair Misalignment Under Superimposed Maneuvering Loads

During actual flight operations, an aircraft engine encounters various working scenarios involving superimposed maneuver loads. To facilitate the analysis of maneuver-induced deformations in the floating spline shaft under these multiple scenarios, the misalignment matrix [U] of the spline pair under a unit maneuver load is first computed. It is then multiplied by the maneuvering load superposition condition matrix [V] to obtain the misalignment matrix [W] of the spline pair under various superimposed maneuvering loads, as shown in Equation (4):

(4) [W]=[U][V],

### 3.2. Misalignment Matrix of the Spline Pair Under Unit Maneuvering Load

The selected unit motorized loads are shown in Table 4, where Ω is the spline shaft rotational speed for the steady-state maximum speed, which is a purely numerical value with no physical significance.

**Figure 5:**
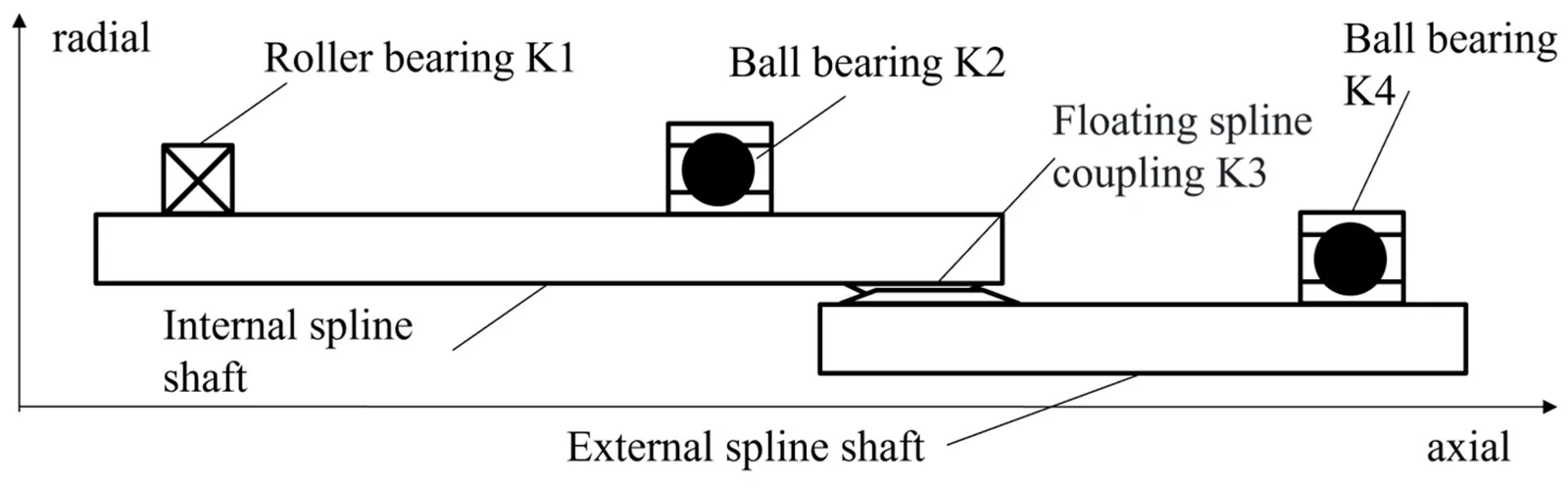
Schematic diagram of a typical floating spline shaft structure for an aero-engine.

The schematic diagram of a typical floating spline shaft structure in an aero-engine is shown in Figure 5. It mainly consists of an internal spline shaft and an external spline shaft. The internal spline shaft is supported on the casing through a roller bearing K1 and a ball bearing K2, while the external spline shaft is supported on the casing through a ball bearing K4. The two shafts realize the transmission of operating torque and mutual support through a floating spline coupling K3.

**Figure 6:**
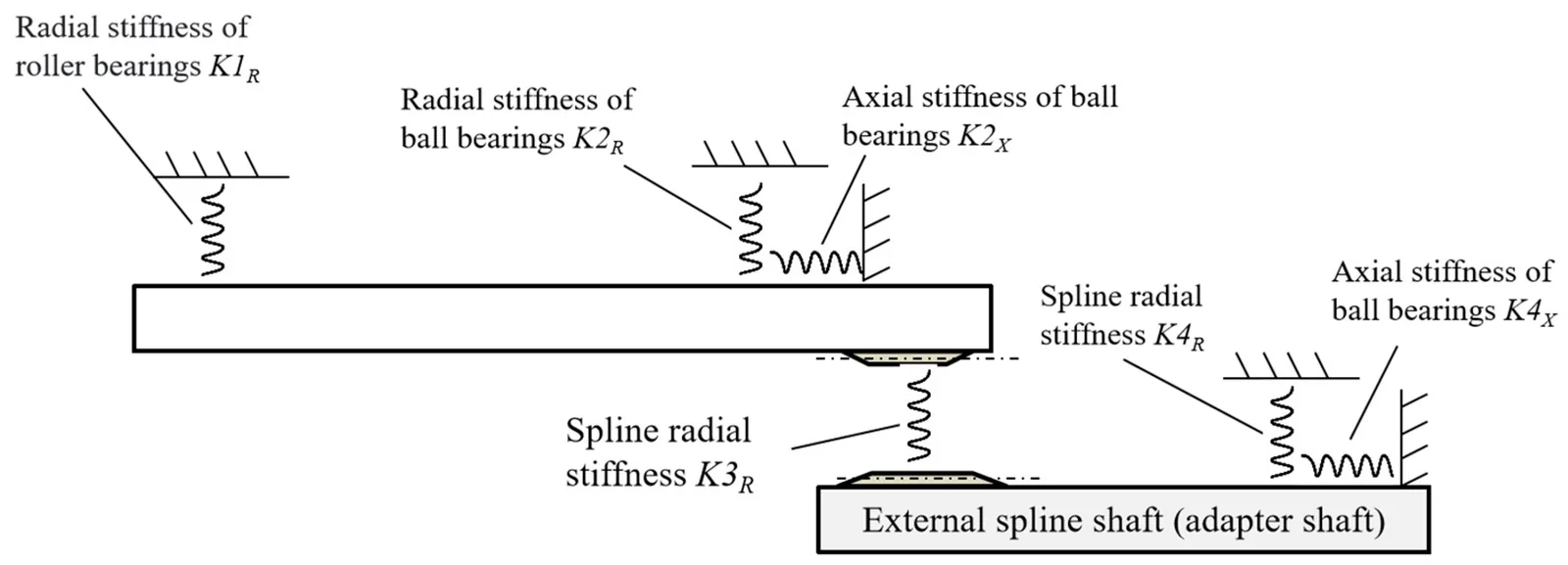
Mechanical model for deformation analysis of spline maneuvering.

**Figure 7:**
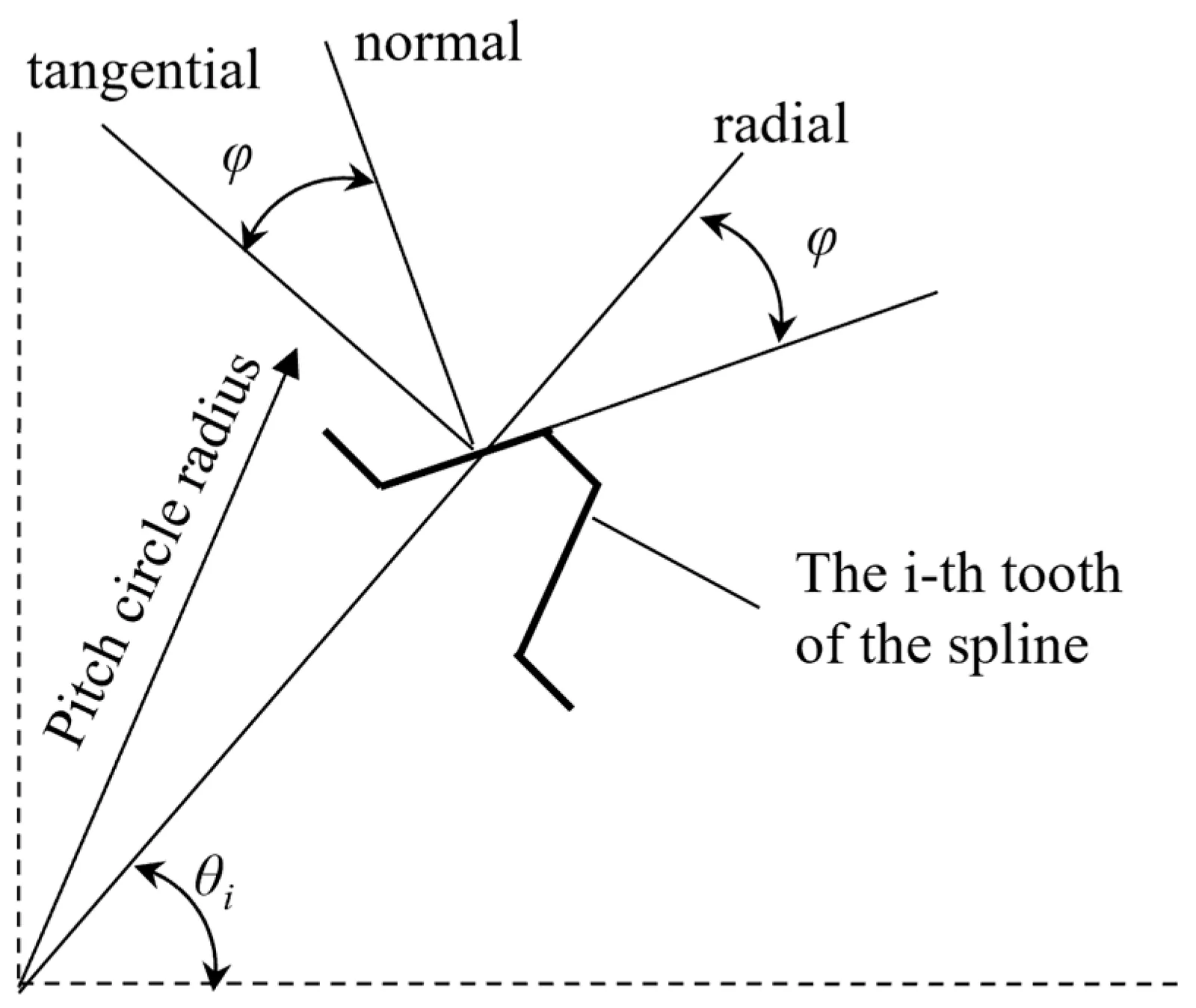
Mechanical model for calculating spline meshing support stiffness.

The maneuvering deformation of the floating spline shaft under unit maneuvering load was analyzed using the SAMCEF 2020.1 finite element software. The mechanical model established for analyzing the maneuver deformation of the floating spline is shown in Figure 6. A radial grounded bearing element was defined at the roller bearing K1. Radial and axial grounded bearing elements were defined at the ball bearings K2 and K4. The spline coupling K3 was modeled with an intermediate bearing element simulating radial support between the two components. In the figure, the subscript R denotes radial support stiffness, and the subscript X denotes axial support stiffness. All the above bearing elements can be directly called in the SAMCEF finite element software. Since the object under study undergoes small deformation under maneuvering loads, a linear stiffness model was adopted to approximately represent the support deformation at the bearings under different maneuvering loads, thereby obtaining the deformation of the entire spline shaft. The required splined shaft support stiffness parameters for the calculation are listed in Table 5. The bearing support stiffness is determined by the stiffness of the bearings and the supporting stator casing. The calculation equation for the radial stiffness of the spline [26] is given in Equation (5)

(5) $$K_{t}=\sum_{i=1}^{z}K_{n}\cos^{2}\left(\theta_{i}+\varphi \right),$$

where refer to Figure 7, z is the number of spline teeth; $\theta_{i}$ is the circumferential angle of the contact point of the i-th tooth of the spline located on the radius of the pitch circle; φ is the contact pressure angle of the spline; $K_{n}$ is the normal stiffness of the spline, which can be obtained by first calculating the displacement (i.e., compliance) of the single-tooth model caused by applying a unit load in the normal direction, and then taking its reciprocal.

In the finite element model, apply the unit dynamic loads listed in Table 4, and calculate the misalignment amount of the floating spline under the unit dynamic loads. Collate the calculated data to form the spline pair misalignment matrix [U], as shown in Equation (6):

(6) $$\left[U\right]=\left[\begin{array}{ccc} u_{X}^{X} & u_{X}^{R} & u_{X}^{\theta }\\ e^{X} & e^{R} & e^{\theta }\\ \alpha^{X} & \alpha^{R} & \alpha^{\theta } \end{array}\right],$$

where the data superscript X in the first column of the matrix [U] denotes the misalignment due to unit axial overload, the data superscript R in the second column denotes the misalignment due to unit radial overload, and the data superscript θ in the third column denotes the misalignment due to unit gyroscopic moment; and the data in the first row of the matrix [U] is the axial misalignment, the data in the second row is the radial misalignment, and the data in the third row is the angular misalignment.

### 3.3. Maneuvering Load Superposition Condition Matrix

Based on the motorized overload stacking coefficients listed in Table 3, the motorized load stacking combined conditions matrix [V] is formed as Equation (7):

(7) $$\left[V\right]=\left[\begin{array}{ccc} 0 & 0 & 0\\ 1 & 1 & 10\\ 3.5 & -3.5 & 1.4 \end{array}\right.\;\;\;\;\left.\begin{array}{ccc} 0 & 5.5 & 5.5\\ 10 & 0 & 0\\ -1.4 & 1.4 & -1.4 \end{array}\right],$$

where the data in the first and second columns of the matrix [V] represent the ultimate load superposition coefficients, and the data in the third to sixth columns represent the extreme limit load superposition coefficients; the data in the first row of the matrix [V] is the axial overload coefficients, the second row is the radial overload coefficients, and the third row is the gyroscopic moment overload coefficients.

### 3.4. Misalignment Matrix of the Spline Pair Under Maneuvering Load Superposition

According to Equation (4), the matrix [W] of spline pair misalignment under the superposition of dynamic loads can be calculated, as shown in Equation (8):

(8) $$\left[W\right]=\left[\begin{array}{ccc} u_{X}^{JX1} & u_{X}^{JX2} & u_{X}^{XZ1}\\ e^{JX1} & e^{JX2} & e^{XZ1}\\ \alpha^{JX1} & \alpha^{JX2} & \alpha^{XZ1} \end{array}\right.\;\;\;\;\left.\begin{array}{ccc} u_{X}^{XZ2} & u_{X}^{XZ3} & u_{X}^{XZ4}\\ e^{XZ2} & e^{XZ3} & e^{XZ4}\\ \alpha^{XZ2} & \alpha^{XZ3} & \alpha^{XZ4} \end{array}\right],$$

In the equation, the first and second columns of matrix [W] represent the spline pair misalignment amounts under ultimate load 1 and ultimate load 2, respectively, while the third to sixth columns represent the spline misalignment amounts under limiting load 1 to limiting load 4. The first row of matrix [W] corresponds to the axial misalignment amount, the second row to the radial misalignment amount, and the third row to the angular misalignment amount.

### 3.5. Interference Verification of Spline Pair Fit

The angular misalignment of the spline is limited by the radial float of the floating spline, ${∆}_{R}$. Normally, the design float of the floating spline has the following relationship with the parallel misalignment and angular misalignment, as shown in Equation (9):

(9) $${∆}_{sj}=\frac{\pi L\alpha }{360}+e,$$

where ${∆}_{sj}$ is the design float of the floating spline; L is the contact length of the floating spline.

If ${∆}_{sj}$ is greater than the radial float of the spline ${∆}_{R}$, interference occurs, which restricts the angular misalignment of the spline, in which case the angular misalignment is Equation (10):

(10) $$\alpha_{cr}=\frac{360}{\pi L}\left({∆}_{R}-e\right),$$

where $\alpha_{cr}$ is the spline angular misalignment at the time of interference.

As a result, the spline interference and the angular misalignment after the interference are determined by Equations (9) and (10), which correct the angular misalignment value in the matrix [W].

## 4. Simulation Analysis of Misalignment of Floating Spline Pair Under Dynamic Overload

### 4.1. Finite Element Model

**Figure 8:**
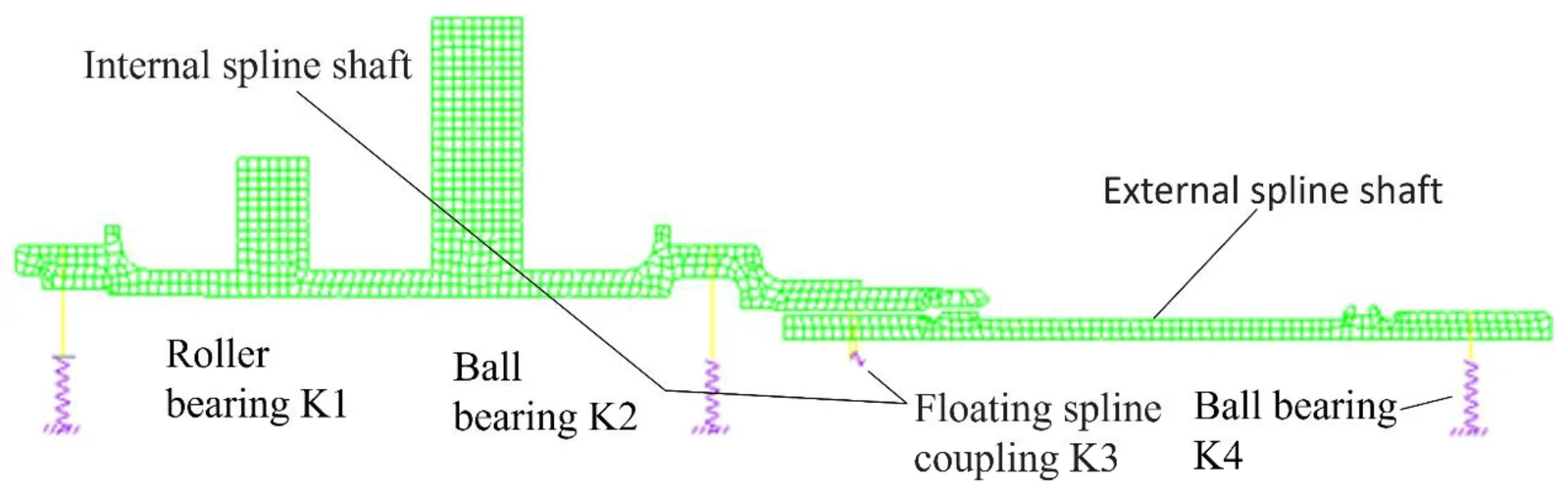
Finite element model of floating involute spline maneuvering deformation for a certain type of engine.

Taking a floating spline of a certain type of engine as an example, the main characteristic structures of the internal and external spline shafts of the floating spline were extracted. The finite element model of the maneuvering deformation of the floating involute spline established using SAMCEF finite element software is shown in Figure 8. The finite element model is a two-dimensional model, the element type is the Fourier element, and the solution module is the static analysis module. Reliable deformation results can still be obtained even when the mesh quantity requirement is not high. Since the focus of this study is on the influence of misalignment caused by maneuvering deformation on contact stress, which belongs to the static/quasi-static category, the influence of dynamic effects on the contact pressure distribution is relatively small. Therefore, static analysis was adopted. The input support stiffness parameters for the dynamic deformation analysis of the spline shaft are listed in Table 6, where κ represents the true value of the support stiffness, which is a pure numerical value without physical meaning. The structural parameters and material parameters of the floating spline are shown in Table 7 and Table 8, respectively.

### 4.2. Calculation Results and Analysis Under Unit Maneuvering Load

**Figure 9:**
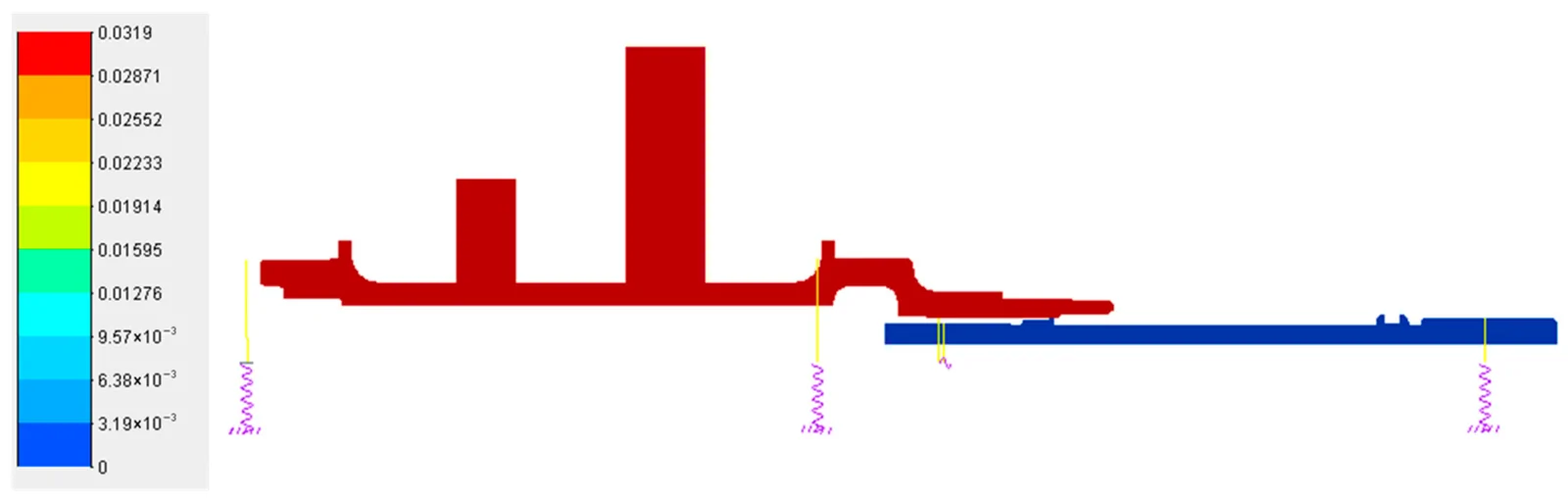
Clouds of motorized deformation of floating spline shaft under axial overload $N_{X}$ = 1 g (mm).

**Figure 10:**
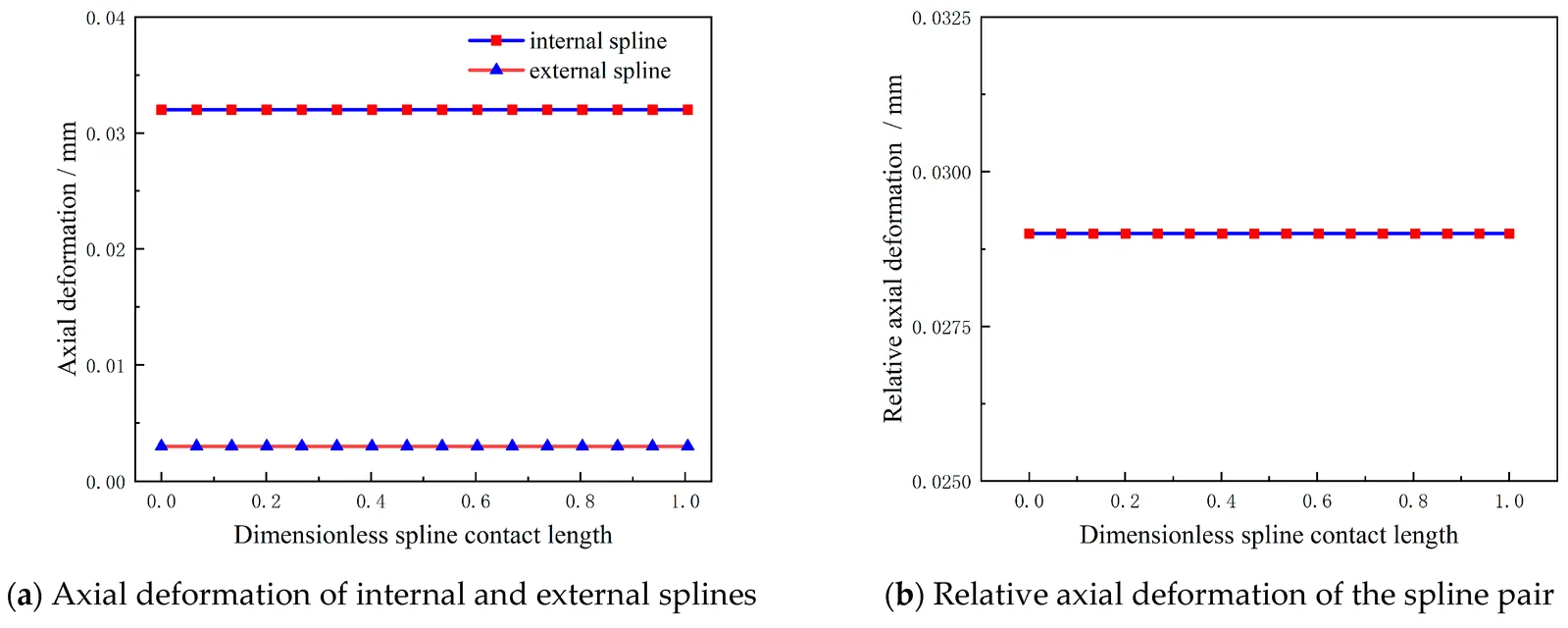
Maneuvering deformation of floating spline pair under axial overload $N_{X}$ = 1 g.

Figure 9 presents the maneuver-induced deformation analysis results of the spline shaft under an axial overload condition of $N_{X}$ = 1 g. It is evident that the deformation primarily manifests as axial deformation. The axial deformation results of the spline pair extracted at this state are shown in Figure 10. The axial deformation does not change with the contact length; that is, the internal and external splines undergo an overall axial misalignment, which can reduce the effective contact length of the spline pair. The results indicate an axial float deformation ($u_{x}$) of the spline pair equal to 0.029 mm. At this point, both the parallel misalignment and angular misalignment of the spline pair are negligible. The axial float deformation is defined as the average value of the relative axial deformation between the spline pair members. A positive value indicates that the axial deformation of the internal spline exceeds that of the external spline, while a negative value signifies the opposite.

**Figure 11:**
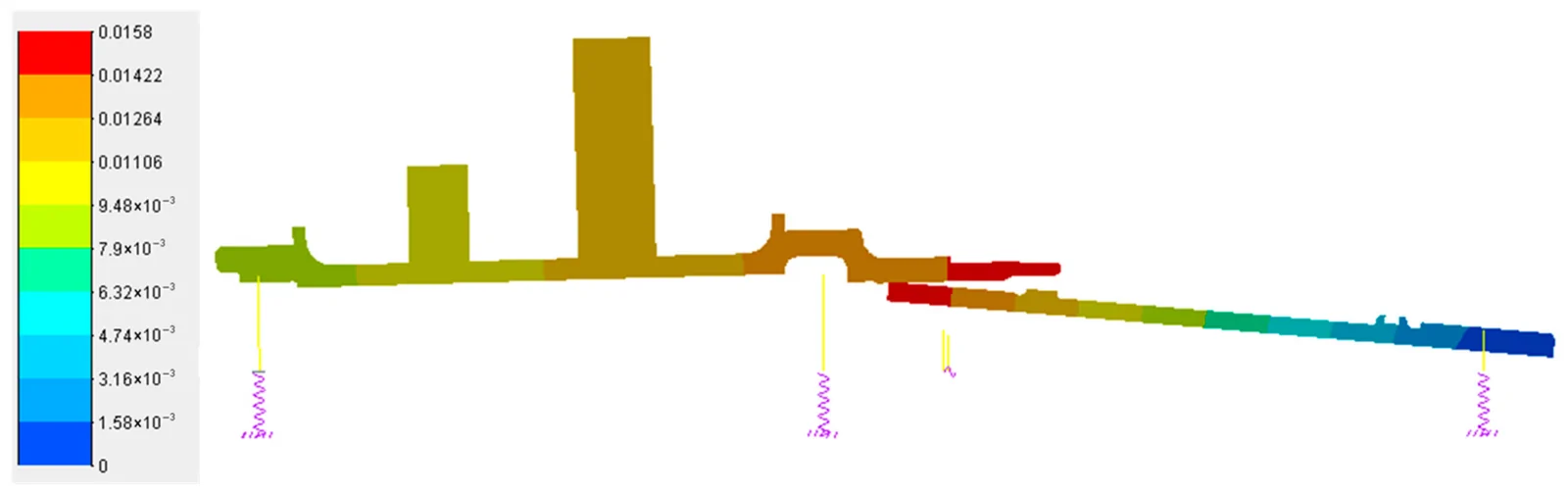
Motorized deformation of floating spline shaft with radial overload $N_{R}$ = 1 g (mm).

**Figure 12:**
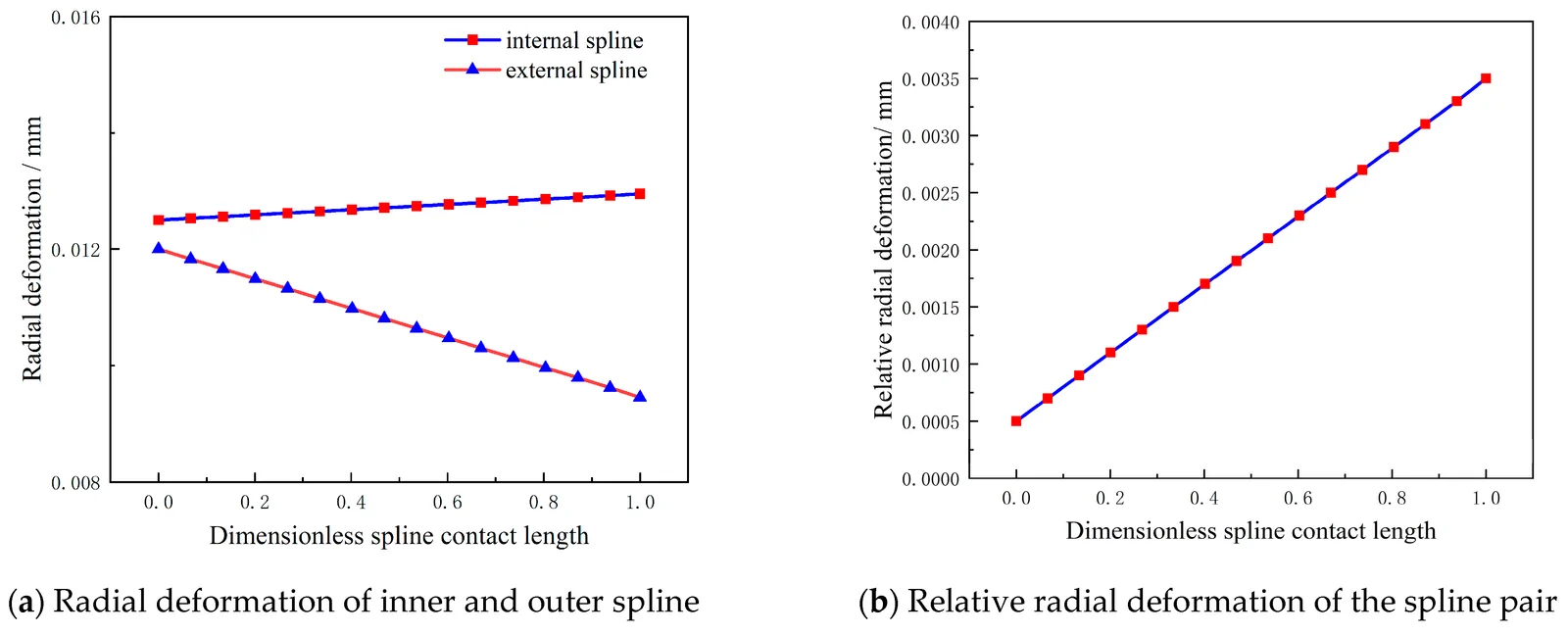
Maneuvering deformation of floating spline pair with radial overload $N_{R}$ = 1 g.

Figure 11 shows the analysis results of the spline shaft’s maneuvering deformation under a radial overload of $N_{R}$ = 1 g. It can be seen that the maneuvering deformation of the spline shaft is mainly radial and angular deformation. The radial deformation results of the spline pair at this time are extracted as shown in Figure 12. The results indicate that the parallel misalignment e of the spline pair is 0.002 mm, and the angular misalignment α is 0.007°. At this time, the axial movement of the spline pair is extremely small and can be ignored. Among them, the parallel misalignment is the average value of the relative radial deformation of the spline pair, and the angular misalignment is the arctangent value of the slope of the relative radial deformation curve of the spline pair. The positive and negative values of the angle are consistent with the positive and negative values of the curve slope.

**Figure 13:**
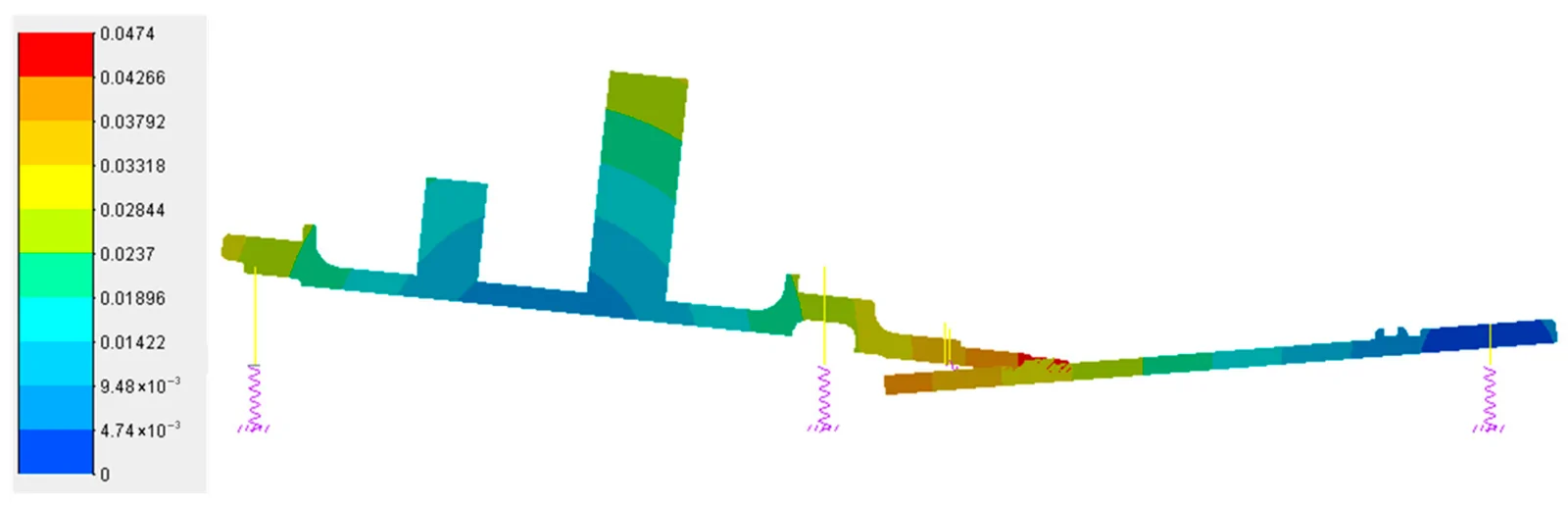
Floating spline shaft maneuvering deformation at gyroscopic moment angular velocity $\omega_{\theta }$ = 1 rad/s (mm).

**Figure 14:**
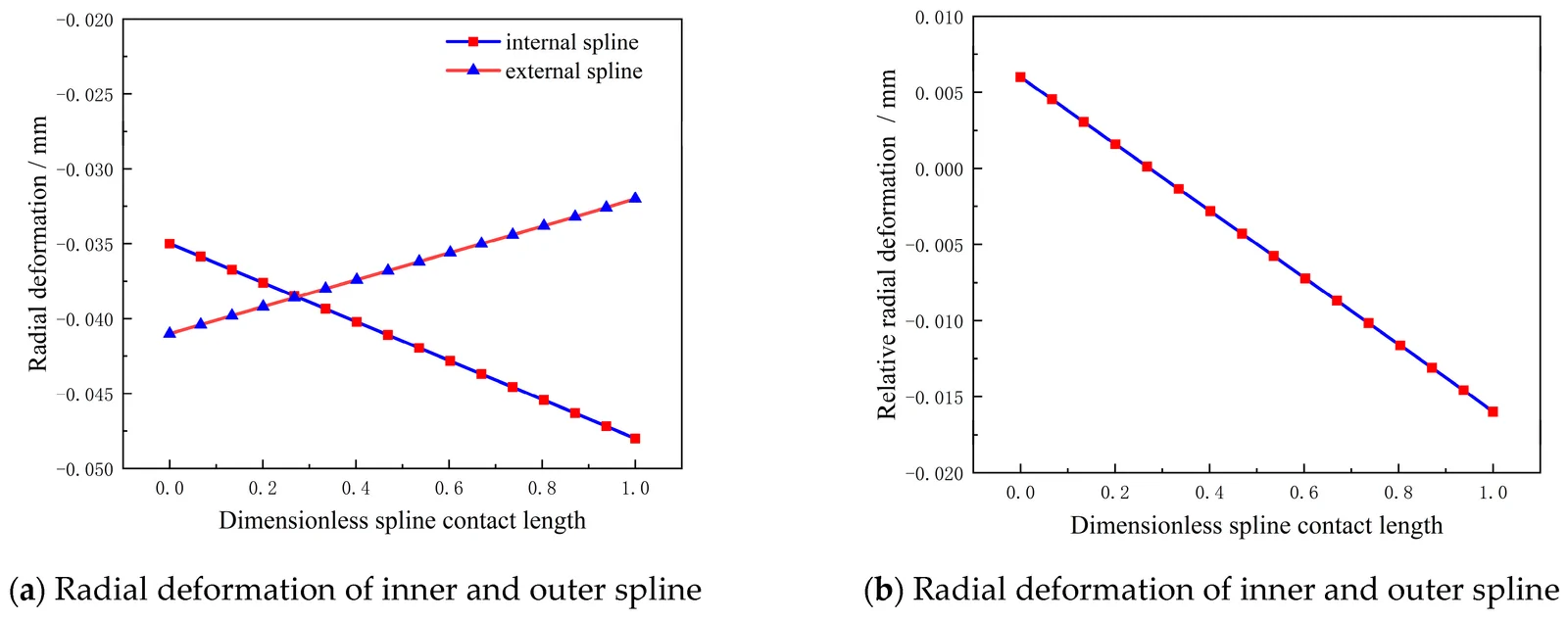
Maneuvering deformation of floating spline sub with gyroscopic moment angular velocity $\omega_{\theta }$ = 1 rad/s.

Figure 13 presents the maneuver deformation analysis results of the spline shaft under a gyroscopic moment $\omega_{\theta }$ = 1 rad/s. It is shown that the primary manifestations of the spline shaft’s maneuver deformation are radial and angular deformation. The radial deformation results of the spline pair extracted at this condition are illustrated in Figure 14. The results indicate a parallel misalignment e of −0.005 mm and an angular misalignment α of −0.045° for the spline pair. The axial float at this point is minimal and negligible. Here, the parallel misalignment is defined as the average value of the relative radial deformation of the spline pair, while the angular misalignment is calculated as the arctangent of the slope of the relative radial deformation curve. The sign of the angular misalignment corresponds to the sign of the curve’s slope.

Different units of maneuvering load under the floating spline misalignment calculation results shown in Table 9, the results show that the engine operating process of the maneuvering load can significantly affect the floating spline misalignment state, in which the axial overload can lead to the floating spline mating surface axial runout, radial overload, gyroscopic moments can lead to the parallel misalignment and angular misalignment, but gyroscopic moments caused by the amount of misalignment is more significant. Therefore, in the design of a floating spline, the maneuvering load significantly affects the parallel misalignment and angular misalignment of a floating spline, especially when the gyroscopic moment is the most significant.

### 4.3. Calculation Results and Analysis Under Superimposed Maneuvering Loads

Table 10 shows the calculation results of floating spline misalignment under the superposition of dynamic overload. The results indicate that under ultimate load 2, the maximum angular misalignment is 0.165°, followed by the parallel misalignment of 0.02 mm; under limited load 2, the maximum parallel misalignment is 0.027 mm, followed by the angular misalignment of 0.133°; under limited load 3 and limited load 4, the maximum axial misalignment is 0.16 mm, while the parallel and angular misalignments are relatively small. The axial misalignment of 0.16 mm is comparable to the allowable assembly error, which has a minor impact on the spline and is within the acceptable range. A large number of studies [11,12,13,14] show that the angular and parallel misalignments of splines significantly change the contact state of spline tooth surfaces, and they are the main factors leading to spline eccentric load, reduced bearing capacity, increased contact stress, contact wear and fatigue failure. Based on the analysis of misalignment and the preliminary screening of contact stress, limit load 2 and restricted load 2 were identified as the most critical operating conditions. Therefore, limit load 2 and restricted load 2 were selected as the key conditions for the spline contact stress analysis. See Table 11 for details.

### 4.4. Interference Verification Results of Spline Pair Fit

**Figure 15:**
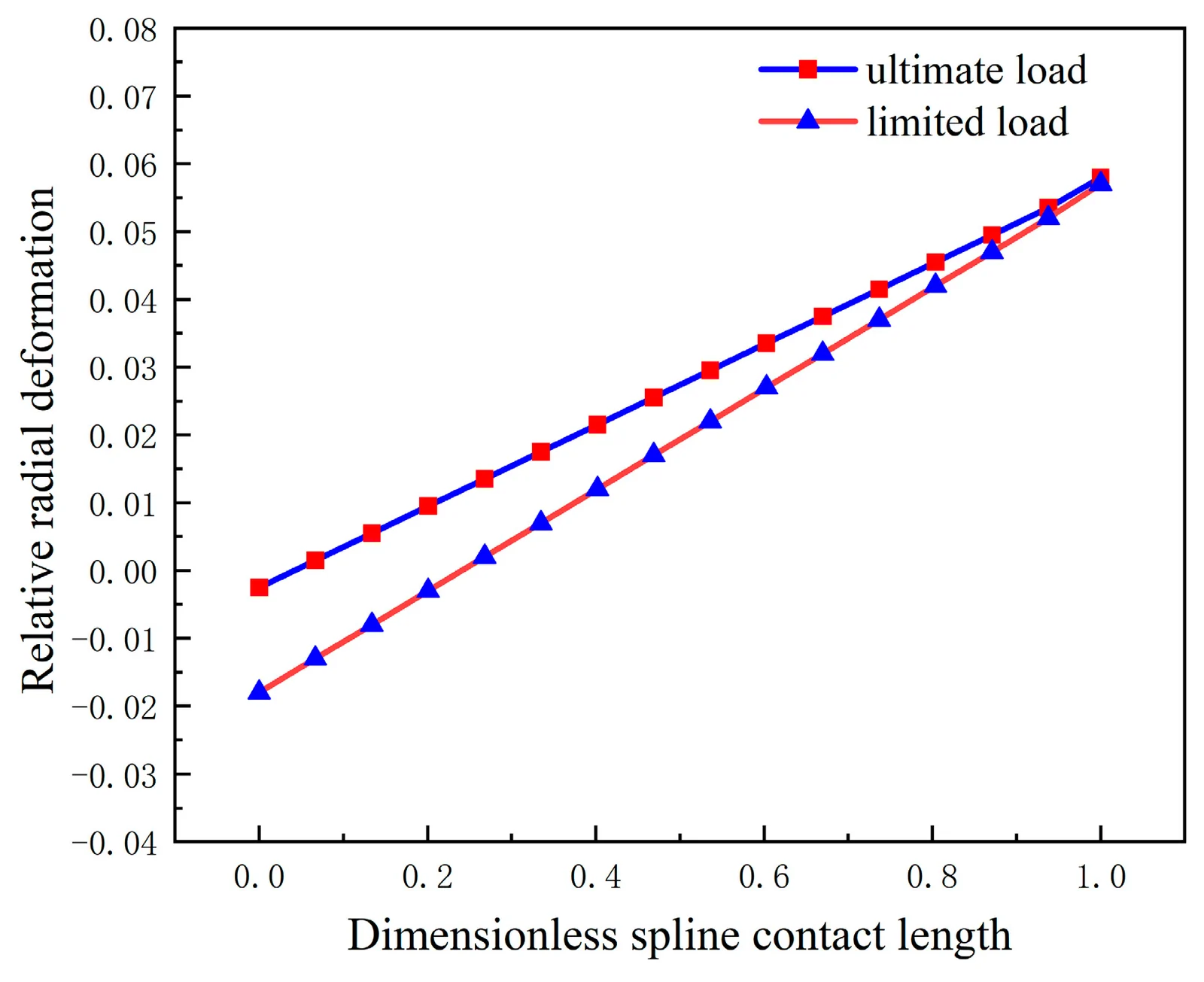
Dynamic deformation of the lower floating spline pair under the superposition of dynamic overload.

Table 12 presents the interference check results for the spline coupling. Figure 15 illustrates the superimposed deformation of the floating spline coupling under maneuver overload conditions. As can be seen from the above, the radial float deformation under both limited load 2 and ultimate load 2 remains below the 0.08 mm threshold mandated by the structural design requirements [20]. This indicates that no deformation interference occurred within the spline coupling. These results confirm that such deformation behavior is consistent with actual conditions during aircraft maneuvering flight.

## 5. Simulation Analysis of Tooth Surface Contact of Floating Involute Spline Pair Under Dynamic Overload

In the previous section, the misalignment behavior of the spline contact pair under dynamic overload conditions was analyzed based on a typical floating spline shaft structure in aero-engines. In this section, the finite element method is employed to separately extract the internal and external spline contact pairs, in order to investigate the influence of misalignment on the tooth surface contact characteristics under combined dynamic overload conditions, and to evaluate the corresponding contact stress and fatigue strength.

### 5.1. Finite Element Model of Spline Tooth Surface Contact

**Figure 16:**
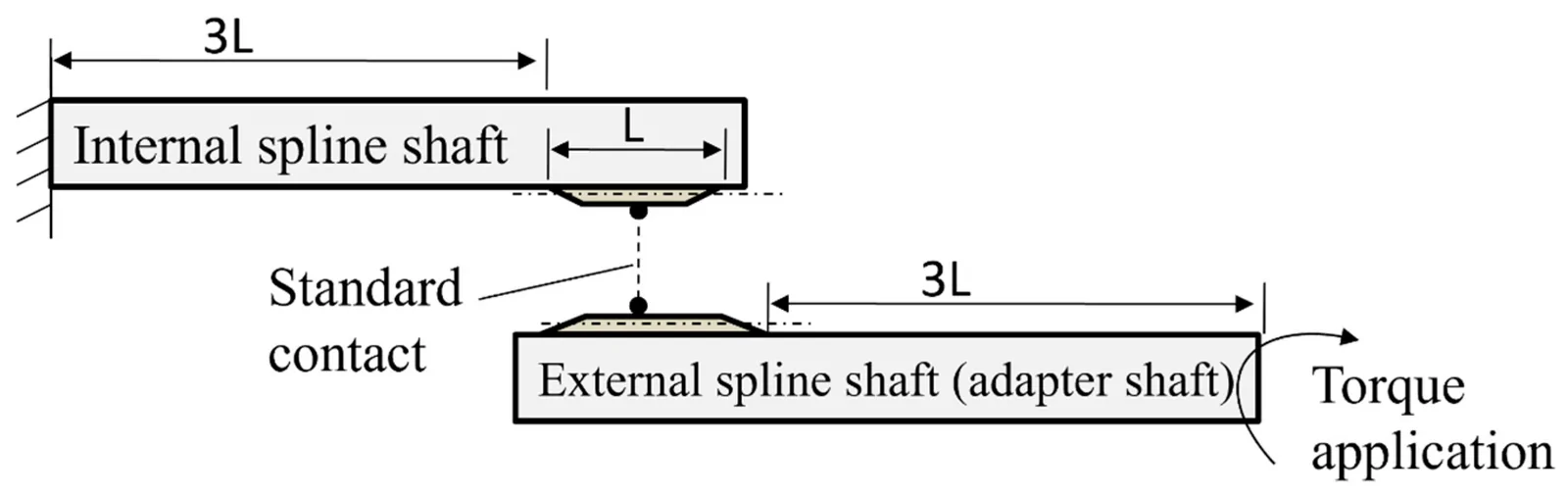
Schematic diagram of loading and constraints for the finite element model of the floating spline.

**Figure 17:**
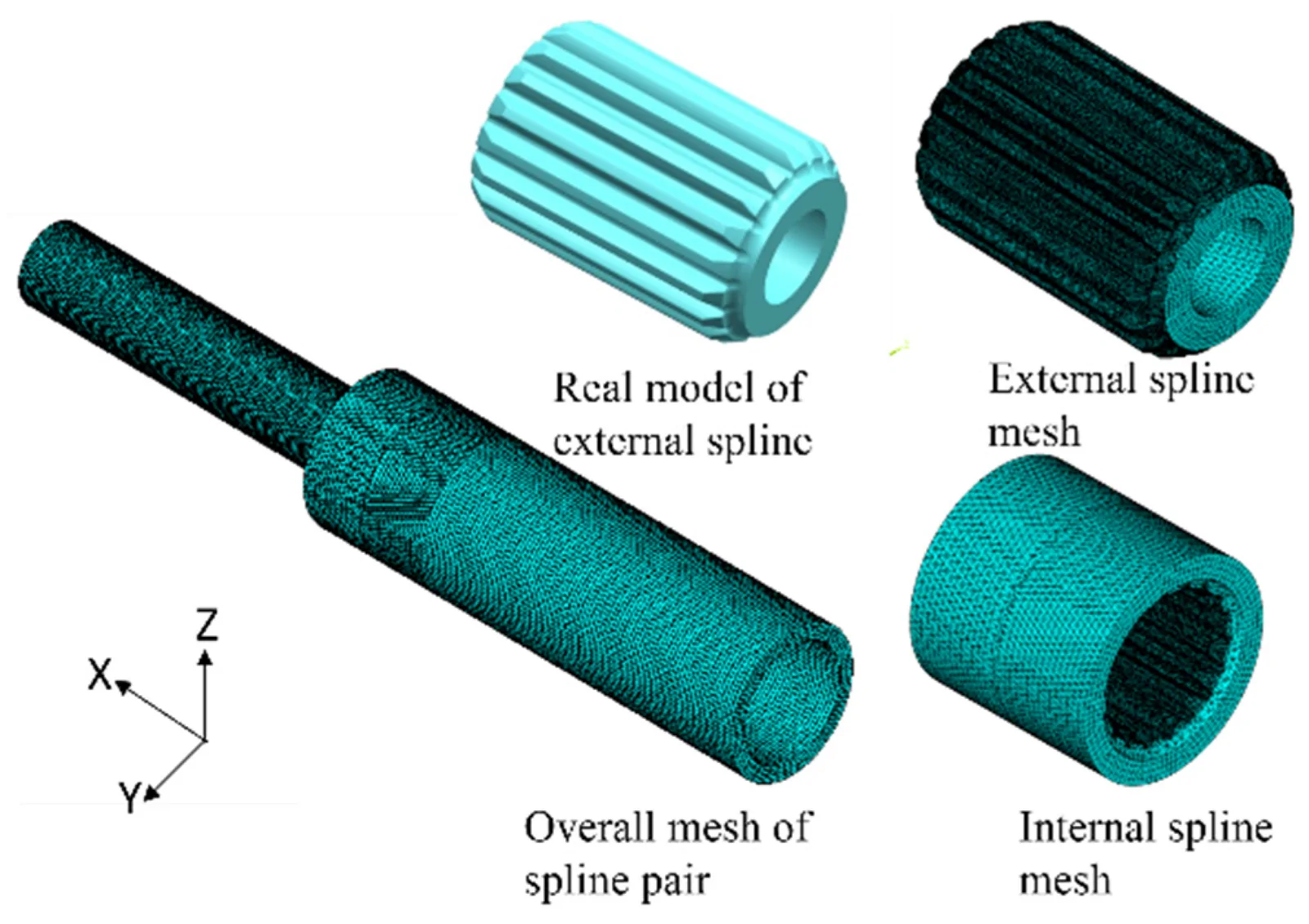
External spline model with rounding and chamfering and mesh refinement.

The WORKBENCH 2020 R1 finite element software was used to conduct the tooth surface contact analysis of the floating spline pair. A finite element model for the tooth surface contact of the spline pair was established with reference to Figure 16. A steady-state maximum torque load was applied to the input end of the external spline, a fixed support constraint was imposed on the output end of the internal spline, and a standard frictional contact was set up on the meshing tooth surfaces of the internal and external splines. To reduce the impact of loads and boundaries on the simulation results of the contact pair, in accordance with Saint–Venant’s principle, the lengths of the spline input shaft and output shaft were set to 3 times the meshing contact length of the spline tooth surfaces. To realistically simulate the meshing behavior of the actual involute spline tooth profile, a real model with geometric features such as fillets and chamfers on the spline tooth surfaces was selected for finite element modeling. Mesh refinement was performed at the tooth surface contact positions and the tooth root fillet areas. The SOLID187 element with mid-side nodes of tetrahedral type was adopted, and a mesh independence study with spline tooth surface mesh sizes ranging from 0.05 mm to 0.4 mm was carried out. Considering both calculation accuracy and efficiency, a mesh size of 0.1 mm was chosen for the tooth surfaces, as shown in Figure 17. The grid independence verification results are shown in Table 13.

**Figure 18:**
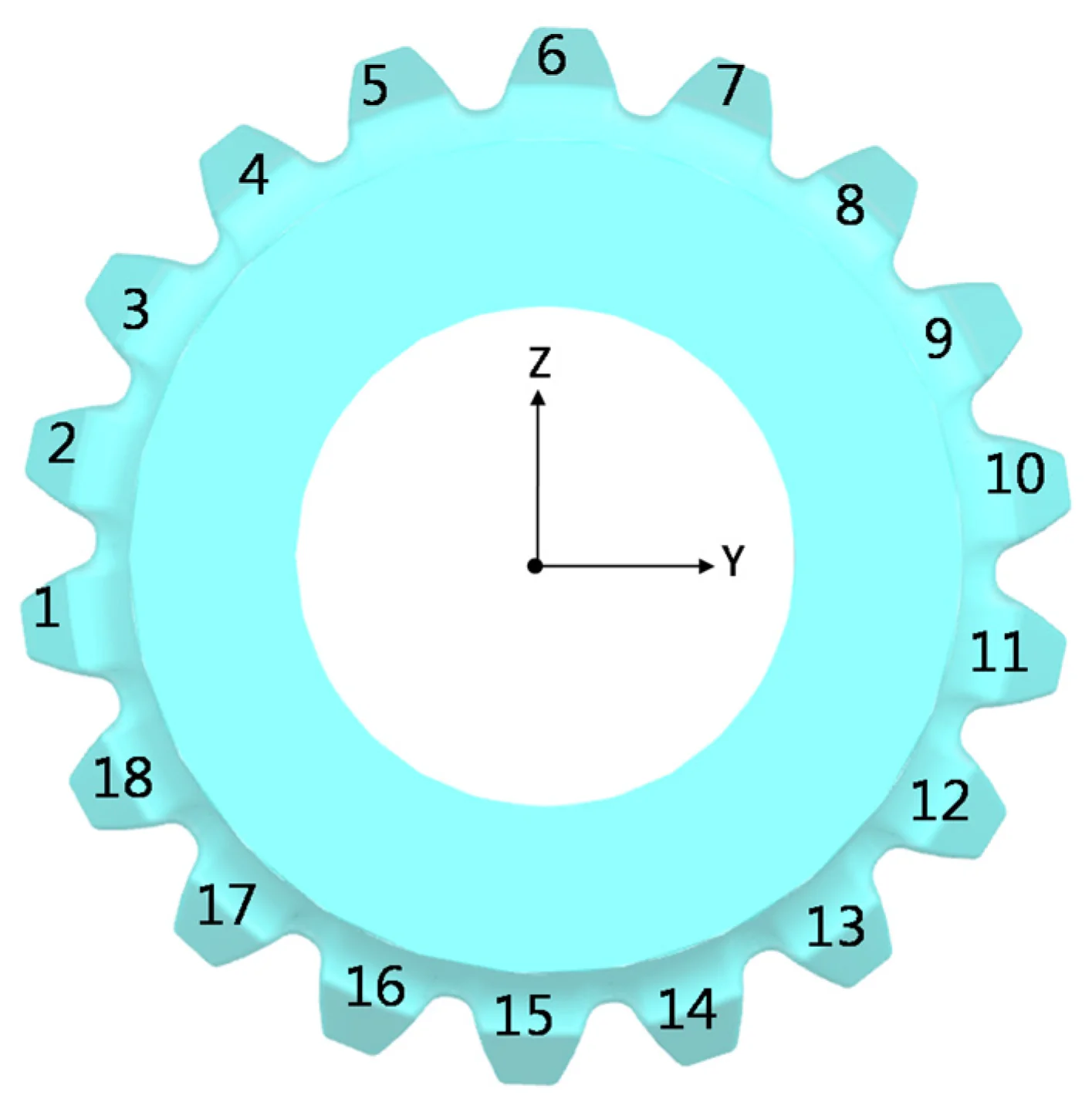
External spline tooth numbering.

The internal and external splines were taken as the contact surface and target surface, respectively. To prevent mesh penetration during the simulation, symmetric contact was established. The contact type was set as standard contact, and the contact algorithm adopted was the augmented Lagrange method. The contact friction coefficient was assumed to be 0.1. Based on engineering experience, this friction coefficient is reasonable because the spline operates in an oil mist or oil-gas environment with self-lubrication. When conducting the tooth surface contact analysis of a floating involute spline pair under maneuvering overload, the misalignment amounts of the floating spline under the ultimate load and limited load specified in Table 11 are artificially set during the assembly process of the internal and external splines. Both the angular misalignment and parallel misalignment occur in the positive Z-direction, as shown in Figure 17. The contact state is checked to ensure that no tooth surface contact interference occurs. To facilitate the explanation of the influence of misalignment on the contact load distribution of each tooth surface, the external spline teeth are numbered according to Figure 18.

### 5.2. Extreme Load Calculation Results and Analysis

**Figure 19:**
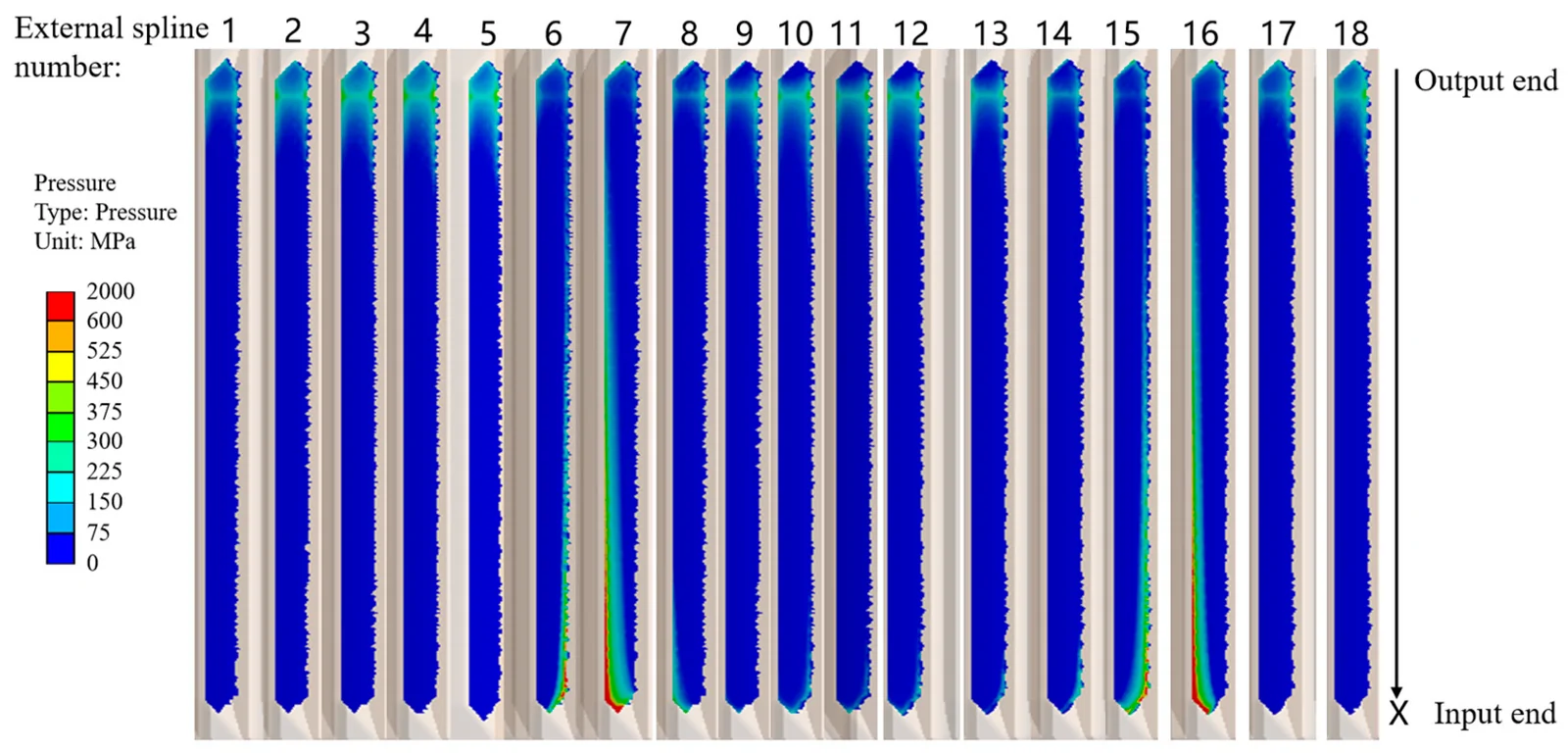
Cloud diagram of contact pressure on the tooth surface of each tooth of the external spline under ultimate load 2.

**Figure 20:**
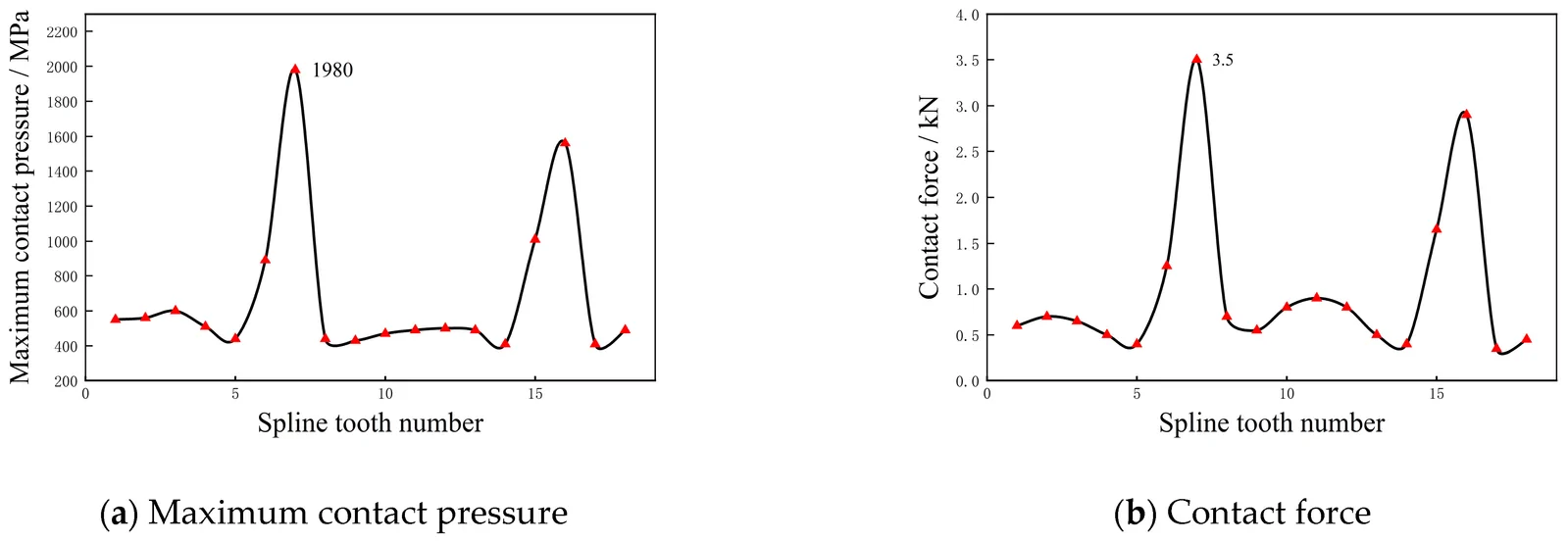
Computation result of each tooth of the external spline under ultimate load 2.

Figure 19 and Figure 20 present the contour maps of maximum contact pressure, as well as the distributions of contact pressure and contact load on each tooth surface of the external spline under ultimate load 2. Under this loading condition, both angular and parallel misalignments occur in the positive direction of the Z-axis. As shown in Figure 19, relatively high stresses are observed at the input end of teeth No. 6, 7, 15, and 16, accompanied by pronounced stress concentration. These locations correspond to the positive Z-axis direction, where angular and parallel misalignments occur, as well as the opposite negative Z-axis direction. In contrast, for the remaining teeth, higher stresses are mainly distributed near the output end, but not at the outermost edge. Figure 20 further demonstrate significant stress concentration and non-uniform load distribution in the spline. The highest contact pressure and meshing forces are concentrated on teeth No. 6, 7, 15, and 16. Among them, tooth No. 7 exhibits the maximum contact stress, reaching 1980 MPa, and sustains the highest contact load of 3.5 kN. From a mechanistic perspective, angular misalignment reduces the effective contact area of the spline, thereby intensifying stress concentration. In contrast, parallel misalignment decreases the backlash of the spline pair, causing the tooth with the minimum backlash to engage first and carry the highest load. This critical region is located in the positive Z-axis direction, where parallel misalignment occurs, followed by the negative Z-axis direction, corresponding to teeth No. 7 and 16, respectively, in the present results.

### 5.3. Limit Load Calculation Results and Analysis

**Figure 21:**
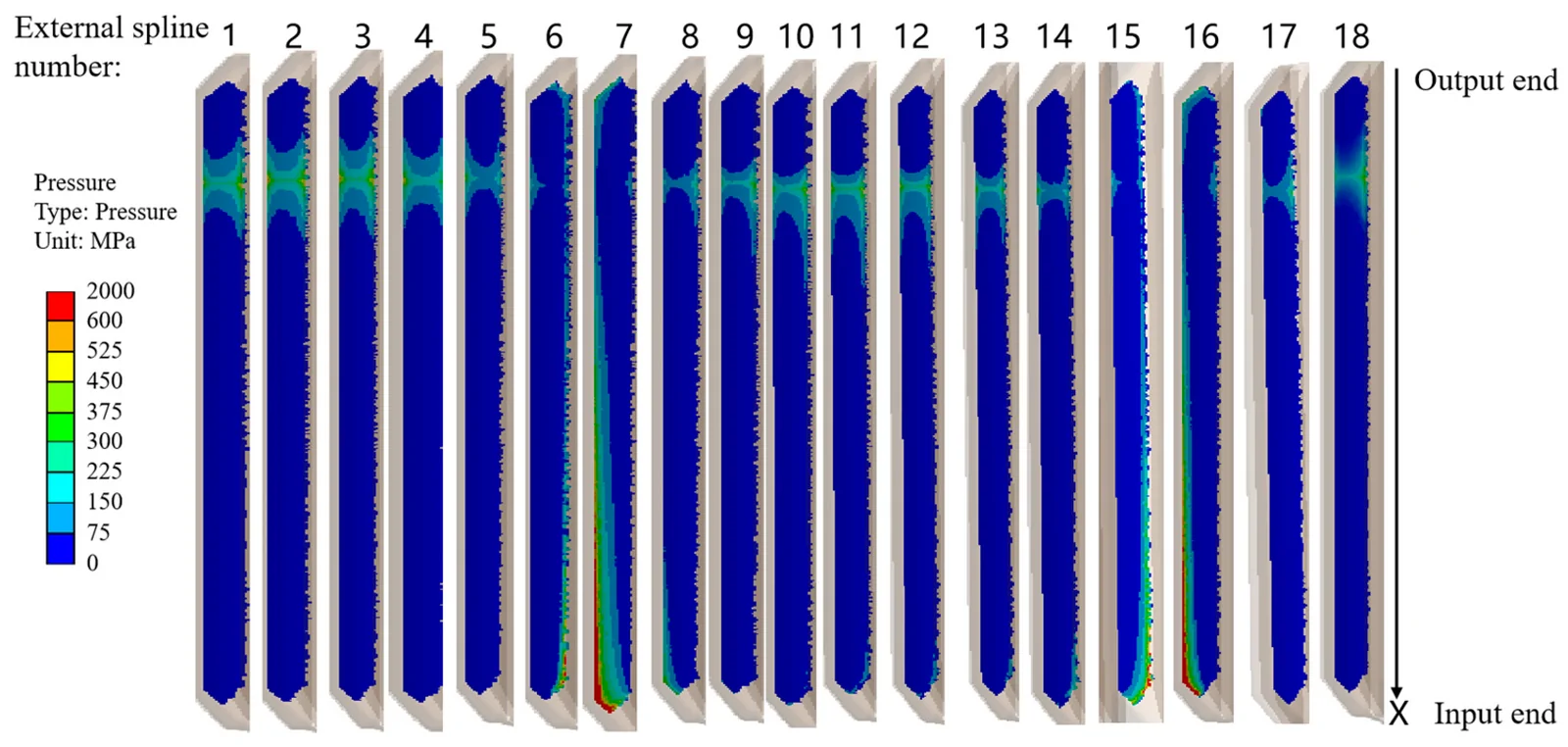
Cloud diagram of tooth surface contact pressure of each tooth of external spline under limited load 2.

**Figure 22:**
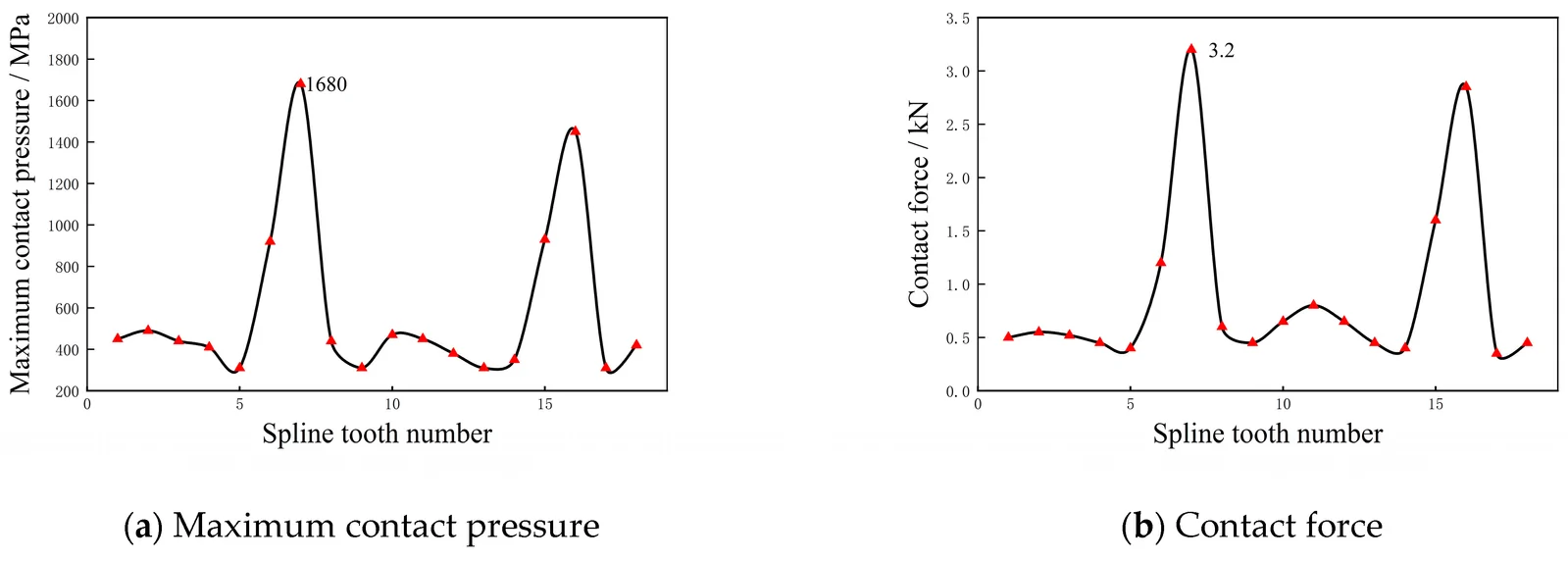
Computation result of each tooth of external spline under limited load 2.

Figure 21 and Figure 22 present the contour maps of maximum contact pressure, as well as the distributions of maximum contact pressure and contact load on each tooth surface of the external spline under limit load 2. Under this loading condition, both angular and parallel misalignments occur in the positive direction of the Z-axis. As shown in Figure 21, relatively high stresses are observed at the input end of teeth No. 6, 7, 15, and 16, with pronounced stress concentration. These locations correspond to the positive Z-axis direction, where angular and parallel misalignments occur, as well as the opposite negative Z-axis direction. In contrast, higher stresses on other teeth are mainly distributed near the output end. Figure 22 further demonstrate significant stress concentration and non-uniform load distribution in the spline. The highest contact pressure and meshing forces are concentrated on teeth No. 6, 7, 15, and 16. Among them, tooth No. 7 exhibits the maximum contact stress, reaching 1680 MPa, and sustains the highest contact load of 3.2 kN. From a mechanistic perspective, angular misalignment reduces the effective contact area of the spline, thereby intensifying stress concentration. In contrast, parallel misalignment decreases the backlash of the spline pair, causing the tooth with the minimum backlash to engage first and carry the highest load. This critical region is located in the positive Z-axis direction, where parallel misalignment occurs, followed by the negative Z-axis direction, corresponding to teeth No. 7 and 16, respectively, in the present results. At the same time, the regions with higher tooth surface contact stress often exhibit greater contact friction forces. It can therefore be inferred that after a period of operation, wear of the spline is most likely to occur first at these local positions.

Load sharing factor (LSF): Defined as the ratio of the contact load carried by a specific tooth to the average tooth load. For ultimate load 2, the maximum LSF reaches 2.35 (tooth No. 7). Percentage of total load carried by the most loaded teeth: The three most loaded teeth (Nos. 67, and 16) together carry 42% of the total transmitted load under ultimate load 2, despite representing only 16.7% of the total teeth. Contact ratio variation: Due to misalignment, the effective contact ratio decreases from the design value of 1.8 to approximately 1.2–1.4, indicating reduced load sharing.

### 5.4. Contact Stress Evaluation

Considering that the circumferential tooth-by-tooth load distribution of the spline changes during maneuvering deformation, each rotation subjects the spline to one load cycle. Fatigue life assessment is therefore required. In accordance with the requirements described in Section 2 regarding maneuvering overload usage, the floating spline must be capable of long-term service under limit load conditions and short-term operation under ultimate load conditions. The restricted load is the load borne by the spline during long-term operation, and the fatigue strength should be evaluated according to the infinite life criterion, as shown in Equation (11):

(11) $$\frac{\sigma_{Hlim}}{K_{xz}\sigma_{xz}}\geq 1,$$

where $\sigma_{Hlim}$ is the contact fatigue limit of the floating spline, which is related to the gear material and surface processing state, and is usually measured by experiments; $\sigma_{zx}$ is the maximum contact pressure under the action of the spline-limited load maneuvering deformation; $k_{xz}$ is the spline long-term working wear life coefficient, which is generally taken as 0.8~0.9 according to engineering experience [28].

The limit load is the extreme load borne by the spline for a short period of time, and it should be evaluated according to the static strength criterion, as shown in Equation (12):

(12) $$\frac{\sigma_{Hlim}k_{NT}k_{w}}{\sigma_{jx}}\geq 1,$$

where $\sigma_{jx}$ is the maximum contact pressure under the action of the spline-limited load maneuvering deformation; $k_{NT}$ is the static strength contact life coefficient. For structural steel, quenched and tempered steel, and carburized and quenched carburized steel, $k_{NT}$ = 1.6 [29]; for nitrided steel (after nitriding), quenched and tempered steel, and carburized steel, $k_{NT}$ = 1.3 [29]; for quenched and tempered steel and carburized steel after nitrocarburizing, $k_{NT}$ = 1.1 [29] $k_{w}$ is the tooth surface work hardening coefficient, with $k_{w}$ = 1.2~1.0 [29].

Based on Equations (11) and (12), the evaluation results are shown in Table 14. The contact fatigue limit is taken as 1470 MPa, which is determined based on the contact fatigue test data of the carburized and quenched layer of the material and the calculation equation for the contact fatigue strength of carburized steel specified in HB/Z 84.2-1984 [29]. The results indicate that the long-term working contact fatigue strength of the floating spline under the limited load working scenario does not meet the design requirements, while the short-term working of the floating spline under the limit load working scenario meets the strength design. Therefore, it is suggested that the spline parameters need to be optimized during the design of the floating spline to meet the long-term working requirements.

## 6. Conclusions

**Figure 23:**
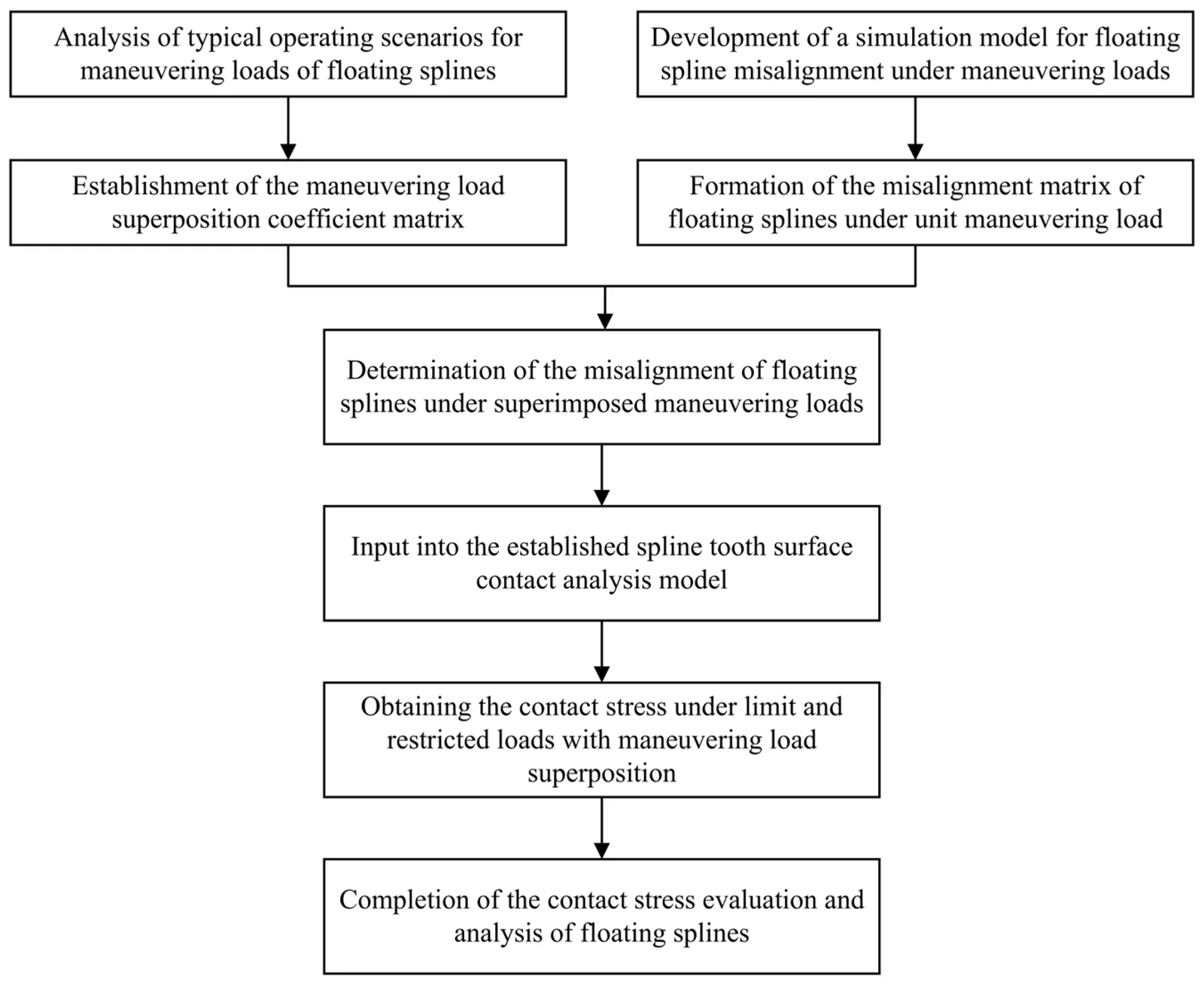
Flowchart of quantitative analysis of floating spline misalignment under the superposition of multiple maneuvering loads.

The main conclusions of this study are as follows:

(1) Considering the coupling effect between maneuvering deformation and misalignment occurring in the actual working state of floating splines, a quantitative analysis method for floating spline misalignment under the superposition of multiple maneuvering loads is established, as shown in Figure 23. This method can effectively evaluate the combined influence of axial overload, radial overload, and gyroscopic moment on the misalignment state of the spline pair.

(2) The analysis of misalignment in the floating spline under combined maneuvering overload conditions shows that, under ultimate load 2, the maximum angular misalignment reaches 0.165°, followed by a parallel misalignment of 0.02 mm. Under limit load 2, the maximum parallel misalignment is 0.027 mm, followed by an angular misalignment of 0.133°. Under both limit load 3 and limit load 4, axial misalignment becomes dominant, reaching a maximum value of 0.16 mm, while parallel and angular misalignments remain relatively small. Notably, the axial misalignment of 0.16 mm is comparable to the allowable assembly tolerance.
(3) The contact stress analysis of the floating spline pair under more severe ultimate and limit loading conditions reveals pronounced stress concentration and non-uniform load distribution. Higher contact pressure and meshing forces are observed on teeth No. 6, 7, 15, and 16, which are located near the positive and negative directions of the Z-axis, corresponding to regions of parallel misalignment. The maximum contact stress on the tooth surface reaches 1980 MPa under ultimate loading and 1680 MPa under limit loading. In these regions with relatively large contact pressure, the friction force is also greater, causing wear of the spline to occur first at these local positions.
(4) According to the load usage requirements and the infinite life and static strength design method, the evaluation results show that the long-term working contact fatigue strength of the floating spline of a certain type of engine under the restricted load does not meet the design requirements (safety factor 0.97), and the spline parameters need to be optimized. However, the short-term working contact strength under the limit load meets the design requirements (safety factor 1.19). The analysis method proposed in this paper can provide a theoretical reference for the misalignment design and long-term working capability improvement of aviation floating splines.

## Figures and Tables

**Table 1:** Types and symbols of misalignment of floating spline pair.

Types of Spline Misalignment	Symbol
Axial misalignment/mm	$u_{X}$
Parallel misalignment/mm	e
Angular misalignment/°	α

**Table 2:** Overload types and symbols.

Type of Dynamic Load	Symbol	Remarks
Axial overload factor/g	$N_{X}$	—
Radial overload factor/g	$N_{R}$	Including vertical and horizontal overloads
Yaw rate/(rad/s)	$\omega_{\theta }$	Including vertical and horizontal yaw
Spin angular velocity/(rad/s)	ω	—
Gyroscopic moment (N·m)	$M_{\theta }$	—
Axial mass inertial force (N)	$F_{X}$	—
Radial mass inertial force (N)	$F_{R}$	—

**Table 3:** Motorized overload stacking factors for typical operating scenarios with floating splines.

Working Scenario	$N_{X}$/g	$N_{R}$/g	$\omega_{\theta }$ (rad/s)
Ultimate load 1	0	1	3.5
Ultimate load 2	0	1	−3.5
Limit load 1	0	10	1.4
Limit load 2	0	10	−1.4
Limit load 3	5.5	0	1.4
Limit load 4	5.5	0	−1.4

**Table 4:** Selected values for floating spline unit motorized load.

Type of Dynamic Load	Symbol	Numerical Value
Axial overload factor/g	$N_{X}$	1
Radial overload factor/g	$N_{R}$	1
Angular velocity of gyroscopic moment/(rad/s)	$\omega_{\theta }$	1
Spin angular velocity/(rad/s)	ω	Ω

**Table 5:** Support stiffness parameter table for input in dynamic deformation analysis of spline shaft.

Parameter Name	Parameter Symbol
Radial support stiffness of the splined shaft roller bearing	K1R
Radial support stiffness of internal spline shaft ball bearing	K2R
Axial support stiffness of internal spline shaft ball bearing	K2X
Radial support stiffness of spline connection	K3R
Radial support stiffness of external spline shaft ball bearing	K4R
Axial support stiffness of external spline shaft ball bearing	K4X

**Table 6:** Table of Support Stiffness Parameters Input for Dynamic deformation Analysis of Spline Shaft.

Parameter Name	Parameter Symbol	Numerical Value/(N/mm)
Radial support stiffness of the splined shaft roller bearing	K1R	1.5κ
Radial support stiffness of internal spline shaft ball bearing	K2R	κ
Axial support stiffness of internal spline shaft ball bearing	K2X	κ
Radial support stiffness of spline connection	K3R	35κ
Radial support stiffness of external spline shaft ball bearing	K4R	κ
Axial support stiffness of external spline shaft ball bearing	K4X	κ

**Table 7:** Structural parameters of involute floating splines.

Parameter Name	Parameter Symbol	Numerical Value
Number of teeth	z	18
Module/mm	m	1
Contact pressure angle/(°)	φ	30
Spline contact length/mm	L	26
Normal stiffness of spline/(N/mm)	$K_{n}$	2.2κ
External spline shaft diameter/mm	$D_{0}$	15
Internal spline shaft diameter/mm	$D_{i}$	28
Spline radial floating amount/mm	${∆}_{R}$	≯0.08

**Table 8:** Involute Floating Spline Material Parameters.

Object	Internal Spline	External Spline
Material grade	16Cr3NiWMoVNbE	18Cr2Ni4WA
Elastic modulus/GPa	215	202
Poisson’s ratio	0.33	0.273
Density/(kg/m^3^)	7860	7910

**Table 9:** Calculation results of floating spline misalignment per unit motorized load.

Dynamic Load	Axial Misalignment $u_{x}$ (mm)	Parallel Misalignment e (mm)	Angular Misalignment α (°)
$N_{X}$ = 1 g	0.029	0	0
$N_{R}$ = 1 g	0	0.002	0.007
$\omega_{\theta }$ = 1 rad/s	0	−0.005	−0.045

**Table 10:** Calculation results of floating spline misalignment under the superposition of dynamic overload.

Working Scenario	Ultimate Load 1	Ultimate Load 2	Limit Load 1	Limit Load 2	Limit Load 3	Limit Load 4
Axial misalignment $u_{x}$ (mm)	0	0	0	0	0.16	0.16
Parallel misalignment e (mm)	−0.016	0.020	0.013	0.027	−0.007	0.007
Angular misalignment α (°)	−0.151	0.165	0.007	0.133	−0.063	0.063

**Table 11:** Calculation results of floating spline misalignment under the superposition of the most dangerous dynamic overload.

Working Scenario	$N_{X}$/g	$N_{R}$/g	$\omega_{\theta }$/(rad/s)	Axial Movement $u_{x}$ (mm)	Parallel Misalignment e (mm)	Angular Misalignment α (°)
Ultimate load 2	0	1	−3.5	0	0.020	0.165
Limited load 2	0	10	−1.4	0	0.027	0.133

**Table 12:** Floating Spline Socket Mating Interference Check Results.

Working Scenario	Designed Floating Amount ${∆}_{sj}$/mm	Radial Floating Amount ${∆}_{R}$/mm	Whether There Is Interference
Ultimate load 2	0.058	0.08	No
Limited load 2	0.057	0.08	No

**Table 13:** Grid Independence Verification Table.

Mesh Size	Contact Stress
0.4 mm	857 MPa
0.2 mm	924 MPa
0.1 mm	947 MPa
0.05 mm	951 MPa

**Table 14:** Evaluation Results of Contact Stress of Floating Spline Pair.

Working Scenario	Contact Stress/MPa	Contact Fatigue Limit/MPa	Check Result	Remarks
Limited load 2	1680	1470	$\frac{\sigma_{Hlim}}{K_{xz}\sigma_{xz}}=0.97$, Not satisfied	$K_{xz}$ = 0.9
Ultimate load 2	1980	1470	$\frac{\sigma_{Hlim}k_{NT}k_{w}}{\sigma_{jx}}=1.19,$ Satisfied	$k_{NT}$ = 1.6, $k_{w}$ = 1.0

## Data Availability

The original contributions presented in this study are included in the article. Further inquiries can be directed to the corresponding authors.
